# Synergistic feature selection and distributed classification framework for high-dimensional medical data analysis

**DOI:** 10.1016/j.mex.2025.103219

**Published:** 2025-02-13

**Authors:** D. Dhinakaran, L. Srinivasan, S. Edwin Raja, K. Valarmathi, M. Gomathy Nayagam

**Affiliations:** a Department of Computer Science and Engineering, Vel Tech Rangarajan Dr. Sagunthala R&D Institute of Science and Technology, Chennai, India; b Department of Computer Science and Engineering, Dr. N.G.P. Institute of Technology, Coimbatore, India; c Department of Electronics and Communication Engineering, P.S.R Engineering College, Sivakasi, India; d Department of Computer Science and Business Systems, Ramco Institute of Technology, Rajapalayam, India

**Keywords:** Medical data analysis, Feature selection, Distributed computing, Recursive feature elimination, and Classification, Synergistic Kruskal-RFE Selector and Distributed Multi-Kernel Classification Framework (SKR-DMKCF)

## Abstract

Feature selection and classification efficiency and accuracy are key to improving decision-making regarding medical data analysis. Since the medical datasets are large and complex, they give rise to certain problematic issues such as computational complexity, limited memory space, and a lesser number of correct classifications. In order to overcome these drawbacks, the new integrated algorithm is presented here: Synergistic Kruskal-RFE Selector and Distributed Multi-Kernel Classification Framework (SKR-DMKCF). The innovative architecture of SKR-DMKCF results in the reduction of dimensionality while preserving useful characteristics of the image utilizing recursive feature elimination and multi-kernel classification in a distributed environment. Detailed evaluations were performed on four broad medical datasets and established our performance advantage. The average feature reduction ratio was 89 % for the proposed method, SKR-DMKCF, which can outperform all the methods by achieving the best classification average accuracy of 85.3 %, precision of 81.5 %, and recall 84.7 %. On the efficiency calculations, it was seen that the memory usage is a 25 % reduction compared to the existing methods and the speed-up time was a significant improvement as well to assure scalability for resource-limited environments.

• Innovative Synergistic Kruskal-RFE Selector for efficient feature selection in medical datasets.
• Distributed Multi-Kernel Classification Framework achieving superior accuracy and computational efficiency.

## Graphical abstract

**Figure:**
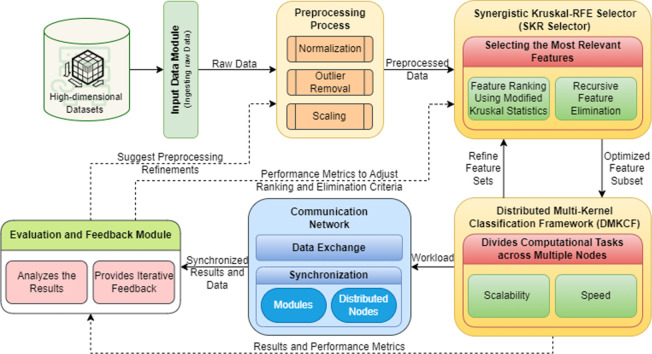

Specifications table

**Table:** 

Subject area:	Engineering
More specific subject area:	Improving decision-making regarding medical data analysis
Name of your method:	Synergistic Kruskal-RFE Selector and Distributed Multi-Kernel Classification Framework (SKR-DMKCF)
Name and reference of original method:	Feature Selection, Distributed Computing, Recursive Feature Elimination
Resource availability:	N*.A.*

## Background

### Introduction

The application of complex computing methods to the healthcare system has led to great discoveries in medical diagnostics, treatment design, and patient supervision. Use of the ML and AI in medical data analysis has been a boon in handling the increasing need for quality and timely healthcare services. Due to the increasing amounts of data collected in the form of EHRs, wearable devices as well as diagnostic tools, there is a rising need to create new methodologies to allow for analysis of this data [1]. These mantras not only assist in coming up with accurate diagnostic results but also assist in the prognosis of disease and working out the best possible treatment options to render. In particular, accumulating medical data is difficult to handle due to its volume, heterogeneity, and complexity, which poses a problem to most computation models [2]. The primary domains impacted by these developments are quite significant given that the selection and classification of the medical dataset's features have been greatly enhanced. The feature selection process plays a critical role in dimensionality reduction whereby the computational cost is reduced together with the number of attributes considered [[3], [4], [5]]. Whereas classification involves the steps of sorting the given data into class labels and class names. Collectively, these processes allow healthcare professionals to gain more quality information about their patients, from huge volumes of data and make better decisions on how to treat those patients.

The medical domain comprises characteristics that separate it from other domains. The first and foremost of the challenges is the reality that dimensions of medical datasets are high, and they may involve ten hundreds or even thousands of features [6]. For example, genomic data or multi-omics datasets are characterized by many features, and the majority of these features are noise or they are interchangeable with other features but for a certain task. This increases the computational expensiveness of the model as well as the poor performance of the Machine Learning models [7]. This is important because finding a subset of features with the highest selectivity and, at the same time, low classification error is one of the most difficult tasks for analyzing data. The other insurmountable obstacle is the issue of comparatively low variability and uneven data distribution inherent in medical datasets. Data samples in the medical field are imbalanced, with some of the classes being rarely or very rarely encountered. This imbalance results in building models that have poor classification of samples from the rare classes, conditions that may be crucial in medicine. Solving this problem calls for higher-level approaches to either balance the dataset or to make sure that the models are not affected by this imbalance. Furthermore, in the medical domain expertise, interpretability, and explainability of the models are required. It can be further noted that though methods like ensemble methods and the operation of deep learning enhance accuracy and are comparatively convincing, their black-box nature challenges their admissibility into the clinic [8]. This is particularly important in clinical decision-making where model predictions must be made clear to clinicians, for example in deciding diagnosis or treatment. Last but not least, the computational cost is always an issue, and this is even more evident, for instance, with distributed and edge environments. There are special cases when medical applications need to analyze data in real-time because delay may produce negative effects. The trade-offs between computational time, memory usage, and scalability remain paramount in developing realistic systems for healthcare solutions.

To cope with the issues of high dimensionality and variability in the data, several feature selection and classification approaches have recently been proposed. Recursive elimination and tree-based techniques are the most popular ones considering the facility for feature selection. Among them, recursive techniques recurrently eliminate less significant features to improve classification. Likewise, to decision trees, feature importance has also been measured in terms of array ensembles, where contributions of the features in the decision trees or forests have been evaluated. Kernel-based methods and ensemble classifiers are considered suitable methods for classification problems. Multi-kernel classifiers and support vector machines are examples of machine learning that formulate arbitrary relations in the data, thus they could be readily applied to medical data [9]. These methods typically incorporate feature selection steps into the classification process in order to strike the best balance between performance and feature set sparseness. To work with a large amount of data, distributed computing paradigms including MapReduce or Spark have also been used in the proposed frameworks. Both of these frameworks partition workloads across the nodes, which greatly cuts down on processing time. However, the current techniques of data filtering are constrained by the following challenges. Unfortunately, many feature selection methods are not best optimized from the perspective of integration for the selection and classification processes [10]. Furthermore, while distributed frameworks are effective, they do not offer the flexibility of data structures for various input data distributions and varying processes. Furthermore, simplicity and practicality are demanding factors in translating interpretable algorithms and optimizing efficiency and performance that justify the use of machine learning models in various healthcare practices.

#### Motivations

*High Dimensionality in Medical Datasets:* It is a common occurrence that there are many features in medical datasets a large number of which are irrelevant or duplicative. This in turn makes the computations more complex and results in poor model performances. Formulating a technique that can enable a healthcare analyst to reduce dimensionality while still retaining much valuable information is crucial if they are to improve on the kind of classification systems that will be available to them.

*Data Imbalance and Variability:* Skewed medical datasets present unique difficulties in addressing the insufficient paradigm replicated threshold. Minority classes signify essential forms, and thus, it becomes always essential to build means by which the classification performance of the classifiers will be guaranteed even with high variability and imbalanced data sets and classes.

*Need for Real-Time Analysis:* It is well understood that medical applications need information that is timely to make efficient decisions. Current approaches experience some challenges of scalability especially when working with high dimensional databases. This requires fast algorithms that can solve the problem using real-time information in order to be utilized in clinical practice.

*Interpretability and Trustworthiness:* A model that needs transparent and clear outputs is important for clinical adoption by healthcare professionals. While previous strategies used in similar issues focus on precision, they fail to provide effective readability. There is a requirement for strategies that perform will be outstanding but are also transparent to assist the professionals in decision-making to gain their confidence.

*Scalability and Adaptability:* In the medical datasets, the size and elaboration are growing, and thus require more scalable methods. Current methods do not integrate well with distributed or edge computing conditions. To help such frameworks be useful for practical employment in healthcare, targeted at data sets of varying sizes and characteristics, it is crucial to encourage the enhancement of scalability and flexibility of the methods used.

#### Objectives

*Develop an Efficient Feature Selection Method:* Develop an ideally efficient feature selection technique that reduces the dataset dimension considerably, and retains the most valuable features. This makes it easier for computations and also improves the classification of other medical data sets.

*Improve Classification Accuracy and Robustness:* Establish a classification model that would justify high levels of accuracy and precise and specific results with less sensitivity to issues of data imbalance and variability. The goal is to achieve better performance than existing approaches in addressing various healthcare issues.

*Enhance Computational Efficiency:* Efficiency of operations by enhancing the distributed architecture and make feature selection and classification integrated and interdependent. Its real-time applicability and scalability characteristics are valuable when coping with large-scale medical datasets.

*Integrate Interpretability into the Framework:* Propose explainable corrective techniques integrated into the feature selection and classification process where in addition to recommending high-performing solutions, there should be reasonable explanations as to why specific decisions were made.

*Validate Across Diverse Medical Datasets:* Experiment on several medical datasets with a diverse set of experiments to demonstrate the practicability of the proposed framework. The idea is to show that it is possible to achieve better performance with every consecutive experiment, on common as well as more complex datasets.

To tackle these challenges, we design a new framework called Synergistic Kruskal-RFE Selector and Distributed Multi-Kernel Classification Framework (SKR-DMKCF) in this work. We propose a unified pipeline for feature selection and classification where the feature selection step is performed with the help of the Kruskal-RFE algorithm. Such an approach allows chosen features to be enhanced for the subsequent classification task in terms of time while increasing its precision. The classification phase employs a distributed multi-kernel framework where several kernel functions are incorporated to characterize the data because it consists of other non-linear relationship attributes. This is because through the distribution of the computations to nodes our method has been able to realize great cuts on the sizes of computations and memory required in medical big data sets. The following sections of this study provide detailed analyses of other important areas of the research. Section 2 focuses on previous studies where the progress and the existing deficit in the feature selection techniques and classification frameworks are discussed. In section 3, the author gives a detailed account of the proposed SKR Selector and DMKCF, explaining what was designed and the originality therein. Section 4 reviewing the experiment shows how the proposed approach works in view of classification accuracy, feature selection, and computational time in four different medical datasets. Last, Section 5 offers final discussions, lists general progress, and presents a direction for further development and optimization of the proposed framework.

### Related work

There remain a lot of issues in analyzing the medical data because the medical datasets are usually high-dimensional, imbalanced, and noisy. The prediction and diagnosis of diseases in their initial stage including diabetes, Parkinson's, and cardiovascular diseases require powerful techniques that would solve challenges like high dimensionality, missing values, and feature redundancy. Different prior studies have used machine learning and deep learning paradigms in addressing these issues. RFE and other related techniques have been employed for effectiveness procedures commonly applied in feature selection to find good features, reduce dimension, and improve the position of the models. Adding to this SVM, neural networks, and ensemble classifiers with feature selection help to enhance diagnostic accuracy. Also, data preprocessing, data augmentation, and hyperparameters tuning are identified as approaches that have been used to address the issues related to the quality of the data, as well as the efficiency of the algorithms. Other works have also envisioned more complex regimes and new kernel-based approaches to improve both predictive capacity and time efficiency. However, current research draws limitations in various areas such as the precise identification of features, cutting down the computation time, and or improving scalability with other datasets.

In their work, Alalayah et al. [11] presented a method of early diagnosing Parkinson's disease based on the disorders of voice. The approach entails deriving features from voice data and using AI algorithms to categorize the subjects as either healthy or having PD (Parkinson's Disease). These feature evaluation with RFE (recursive feature elimination), data balancing applying SMOTE (synthetic minority oversampling technique), dimensionality reduction applying t-SNE (t-distributed stochastic neighbor embedding), and PCA (principal component analysis). Senan, et al., [12] were involved in the development of a CKD risk that aims at early identification of CKD utilizing machine learning techniques. In the current research data from 400 patients was cleaned and normalized by statistical measures to handle missing values before applying RFE for feature selection. Therefore, they compare the performance of four classifiers: support vector machine (SVM), k-nearest neighbors (KNN), decision tree, and random forest based on the chosen features. In their work, Zhou et al. [13] proposed to classify the modulation types in communication systems using the SVM hyperplanes and cumulant features. Recursive feature elimination (RFE) is used to rank/select features using an independent criterion of how well they separate the modulation types given their linear separability. Balakrishnan et al. [14] introduced a new efficient and accurate predictive technique known as Recursive feature elimination and artificial neural networks (R-ANN) to solve a problem related to Alzheimer's Disease (AD). The concept of RFE is used in the current study to choose important features that will improve computational efficiencies, while a subsequent ANN model helps in accurate disease prediction.

Sabitha et al. [15] make a call for data preprocessing, feature selection, and data augmentation for disease diagnosis. To this end, based also on the PIMA Indian Diabetes dataset, the authors make a comparative analysis of the models employing and not employing preprocessing and look at the effect of the RFE selected features as well as assess the performance of five classification algorithms. The problem of providing risk for cardiovascular diseases in diabetic patients is solved in the paper of Channabasavaraju et al. [16] using the merging of diabetes and heart disease datasets. The survey also presents a new random forest feature selection (RFS) approach to establish the most important features and remove any duplicated features to promote the model's predictive precision. A new feature selection method is proposed for online Arabic handwriting analysis by Meryem et al. [17]. They include copying imposed text and writing a desired text. The study uses RFECV with SVM, decision trees, and random forests to feature selection and then perform classification to detect PD. In the study of lung cancer-related multi-omics datasets, Azman et al. [18] developed a feature selection framework known by support vector machine-recursive feature elimination (SVM-RFE). The integration of genomics, transcriptomics, and epigenomics multi-layered data is also performed, feature subsets are evaluated, and SDAE and VAE classifiers are used to quantify the selected feature subsets. In the area of hyperspectral image analysis, Hassanzadeh et al. [19] propose a GMKL (graph-based multiple kernel learning) that aims at improving feature discriminability. A new block diagonal representation algorithm which detects low-rank graph structures to provide optimum kernel mapping to be used by SVM classification on the Indian Pine dataset and Pavia University dataset is presented.

According to Zhou et al. [20], a new model called deep autoencoder with multiple kernel learning (DAEMKL) for the prediction of MDAs is proposed. The model builds a similarity network of miRNA and disease and then the model makes predictions of novel MDAs through the process of reconstruction error analysis of feature representation. Using the data preprocessing technique, Manoj Kumar et al. [21] developed a supervised DNN using the PIMA Indian Diabetes database to predict diabetes. The importance of features computed is computed from Extra Trees or Random Forest models to measure the accuracy of the classifying model after feature selection. Rajagopal et al. [22] proposed a synthesized hybrid pattern identification model in the healthcare data set with data preprocessing using a state-of-the-art normalization strategy. A decision-making algorithm is used to prioritize variables whereas a specific form of regularization is used for predicting diabetes only. Another work by Chollette et al. [23] making the prediction of diabetes involved the use of feature selection and imputation. Performance measures of classification on the two datasets, namely the PIMA Indian Diabetes dataset and LMCH diabetes dataset are used to confirm the efficiency of the proposed framework. García et al. [24] presented a deep learning pipeline to perform both data augmentation using a VAE and feature augmentation using an SAE. The classifier is done through a CNN with proven efficiency in the PIMA Indian Diabetes dataset. The work of Naz et al. [25] discusses machine learning for early diagnosis of diabetes complications and its importance. The methodology employs a range of algorithms to analyze EHRs and other big data modalities to predict underlying patterns. Huaping et al. [26] developed a deep neural network model to predict the incidence of diabetes and the type of disease. Applying stochastic binary cross entropy as the loss function, diabetes checking is redefined as a classification problem with dropout regularization that yields high accuracy for type 1 and type 2 diabetes cases.

A number of researchers have used machine learning for disease diagnosis and prediction. However, there are some issues that arise in this area. Most of the existing work on this topic is based on particular datasets like the PIMA Indian diabetes dataset, Parkinson datasets and so on which may not be generalizable to different patient populations. Conventional feature selection procedures such as RFE and other combination models however do not sufficiently tackle irregularities in the coherence of the individual features in various diseases. Furthermore, preprocessing procedures and hyperparameters are often dataset-dependent, and most of the works do not have adequate frameworks for merging datasets or dealing with multimodal data. In addition, studies about deep learning models including autoencoders and DNNs have been also conducted but these models ignore the explainability and expansibility for real-world clinical application. Currently, there is no readily available holistic, flexible, and scalable approach to navigate through the complexity of big data often involving datasets of different types, selecting relevant features, and improving the prediction model's performance.

The proposed work aims to fill the gaps as highlighted by proposing an SKR Selector and the DMKCF system. This framework precisely addresses problems related to metrics of high dimensions, including feature redundancy, noise, scalability, and classification efficiency. Employing both advanced features selection methods and distributed classification, our approach offers a sound and extensive solution for modern data-driven application systems. The SKR Selector uses a technique that is a combination of Kruskal-Based Variance Ranking (KVR) and Recursive Feature Elimination (RFE). Due to this method, the selection of informative features is performed taking into account the reduction of noise and irrelevant data impact, and minimization of computational time. The second phase, driven by the DMKCF, adopts distributed multi-kernel learning to improve the accuracy and feasibility of the classification for various high-dimension datasets. All these components add up to form a holistic system where each component increases the computational facilitation and the resilience of the models while also improving the ways through which the models can be understood as well as used in various practical applications. The proposed framework successfully addresses these challenges and adequately fulfills the critical gaps in the selection of features and classification for the high-dimensional data set.

## Method details

### Proposed feature selection and distributed classification framework

**Fig. 1:**
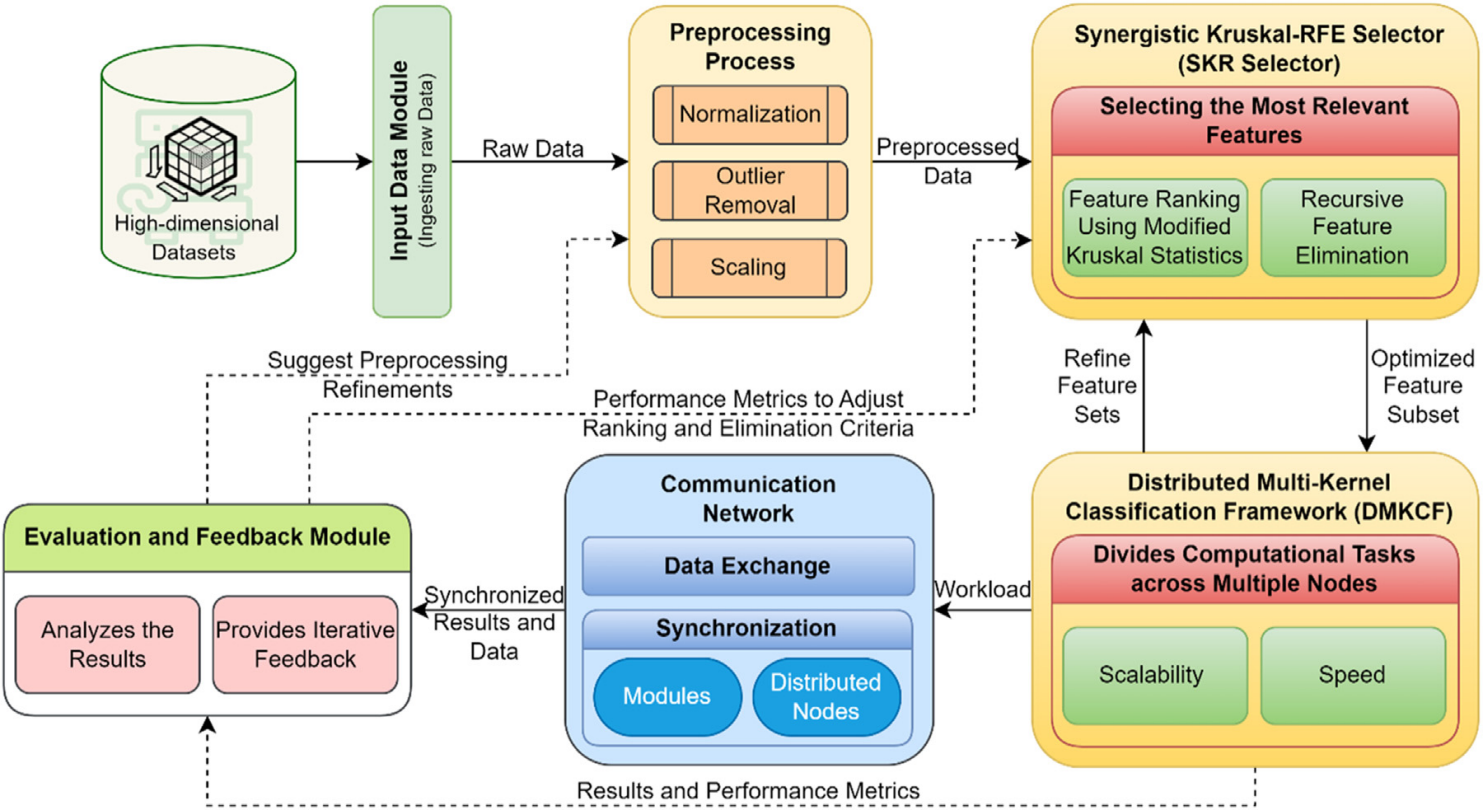
Proposed Feature Selection and Distributed Classification Framework.

The proposed work combines the Synergistic Kruskal-RFE Selector (SKR Selector) and the Distributed Multi-Kernel Classification Framework (DMKCF) to give a well-built system for high-dimensional data analysis and classification. The system is particularly well-aimed to resolve major limitations in feature selection, noise treatment, scalability, and classification accuracy, thus constituting an all-inclusive solution to modern data-orientated applications [27]. The proposed framework jointly integrates the benefits of feature selection methods with distributed and multiple kernel learning methods in terms of computational speed and sturdiness as well as increased accuracy. The first phase of the system is related to feature selection by means of the SKR Selector, which is a hybridized technique of KVR (Kruskal-Based Variance Ranking) and RFE (Recursive Feature Elimination) as shown in Fig. 1. Utility of working with high-dimensional datasets is that some of them may include noisy features or features which are completely unrelated to the task within focus. These features complicate the problem, adding computational load, and decreasing the model interpretability of machine learning models. The SKR Selector addresses this by first enumerating all possible features and then selecting only those with the most informative features.

The SKR Selector begins by sorting features in terms of relative scores and from the variance contributions as well as rank-based metrics. The variance contribution calculates how widely the feature values of the classes are spread and identifies the features that are most differing in the classes. At the same time the rank-based metric derived from the modified Kruskal statistics measures the between-class variability and, therefore, the ranking is precisely ‘‘robust to noise and outliers.” Subsequent to the first round of ranking in the SKR Selector, the algorithm applies the technique of recursive elimination to reduce a step-by-step feature set incrementally. At each step, low-relevance features are eliminated by using classifier-derived importance weights for a given data set. This iterative pruning in a way guarantees the elimination of as many less relevant features as possible while slowly eliminating dimensions. It has been shown that the employment of the objective function that considers relevance, accuracy, and computational cost when selecting features will yield the best results. The result of the SKR Selector phase is therefore a set of features that has been simplified and optimized to contain no duplicated or unnecessary data which will be fed to the classification framework. By excluding some common attributes, this basic chic clearly lowers computational loads and improves the interpretively of future predictive models.

The second phase of the system for predictive modeling uses the DMKCF as its basis. Given the constant growth of the dataset's volume and variety, centralized classification methods commonly encounter feasibility issues in terms of scalability or computational speed. In DMKCF, to address these challenges, a distributed architecture is implemented, and a multi-kernel learning approach is used. DMKCF works in a distributed structure, managing both data and computation load across computational nodes. The architecture of this system also makes it scalable in that it prepares the system for handling large sets of data. They proposed that, in a given system, every node comes into contact with only a portion of the data; this makes the loads on the individual units lighter. The outcome obtained from each node is then combined to construct a global model. To perform the classification in DMKCF, there is the use of multiple kernel functions all in the same system, multi-kernel, for handling linear as well as nonlinear data formats. Besides, the kernels including polynomial, Gaussian, and radial basis functions (RBF), the flexibility of this framework is significant for various data distribution. This results in a multi-kernel strategy improving the discriminative ability of the model and the ability to identify more intricate patterns in the database. Due to the distributed setup of DMKCF, effective model aggregation and optimization methods are required. The framework utilizes kernel alignment and weight optimization strategies to deal with the integration of individual kernels into the final product, thus assuring all kernels of similar reliability, making the final global model highly cohesive. This fusion process also encompasses error-correcting approaches to deal with discrepancies that are caused by distributed computing.

The integration of the two systems: SKR Selector and DMKCF guarantees that it has a rational and coherent process. It starts with the raw data set in their raw formats being pre-processed and the features being selected by the SKR Selector. Here, we continuously rank and exclude features so that this phase yields a high-quality dataset of low dimensionality for classification. After the generation of the refined feature set, it is passed to the DMKCF in which classification is paralleled throughout nodes. The multi-kernel methodology guarantees the inclusion of a wide variety of data properties within the framework, whereas the distributed architecture guarantees high efficiency [28]. The harmony between the feature extracted by SKR Selector and DMKCF means the classification framework runs on a clean and efficient list of features, which improves the predicted results and decreases the computational time.

#### Design goals for SKR selector and DMKCF

The objective of the given method is to improve the classification of distributed data for high dimensions to create a strong foundation for feature selection and accurate, efficient classification of distributed datasets.

1. *Feature Relevance and Optimization:* Prioritize identifying and selecting informative features while minimizing redundancy and noise to enhance computational efficiency and model interpretability.
2. *Scalability and Distribution:* Support data distribution of large datasets which is good for scalability, has less overhead on computational resources and will improve resource usage.
3. *Multi-Kernel Flexibility:* Implement several kernel functions that allow to estimate various linear and non-linear dependencies and avoid the rigid data structure setting.
4. *Robustness to Variability:* Design mechanisms to handle data variability, outliers, and inconsistencies, ensuring reliable and accurate predictions in dynamic environments.
5. *Seamless Integration:* Ensure a cohesive workflow between feature selection and classification phases for streamlined data processing and enhanced end-to-end performance.

#### Synergistic Kruskal-RFE selector (SKR selector) process

The Synergistic Kruskal-RFE Selector (SKR Selector) is a newly proposed framework made for the purpose of improving the prediction accuracy of machine learning models. This approach comprises two major procedures: Kruskal-Based Variance Ranking (KVR) for the selection of features with the highest variance discriminant in distribution syntaxes between classes and Recursive Feature Optimization (RFO) for optimization. The hybrid structure of the SKR Selector provides the possibility to achieve the best computational result and select features that are relevant to the target task most of all. The SKR Selector is developed to address:

1. The identification of the most informative features in high-dimensional datasets.
2. The mitigation of noise and redundancy in feature sets.
3. A computationally efficient feature ranking and elimination process to enhance model accuracy and interpretability.

##### Methodology

**Fig. 2:**
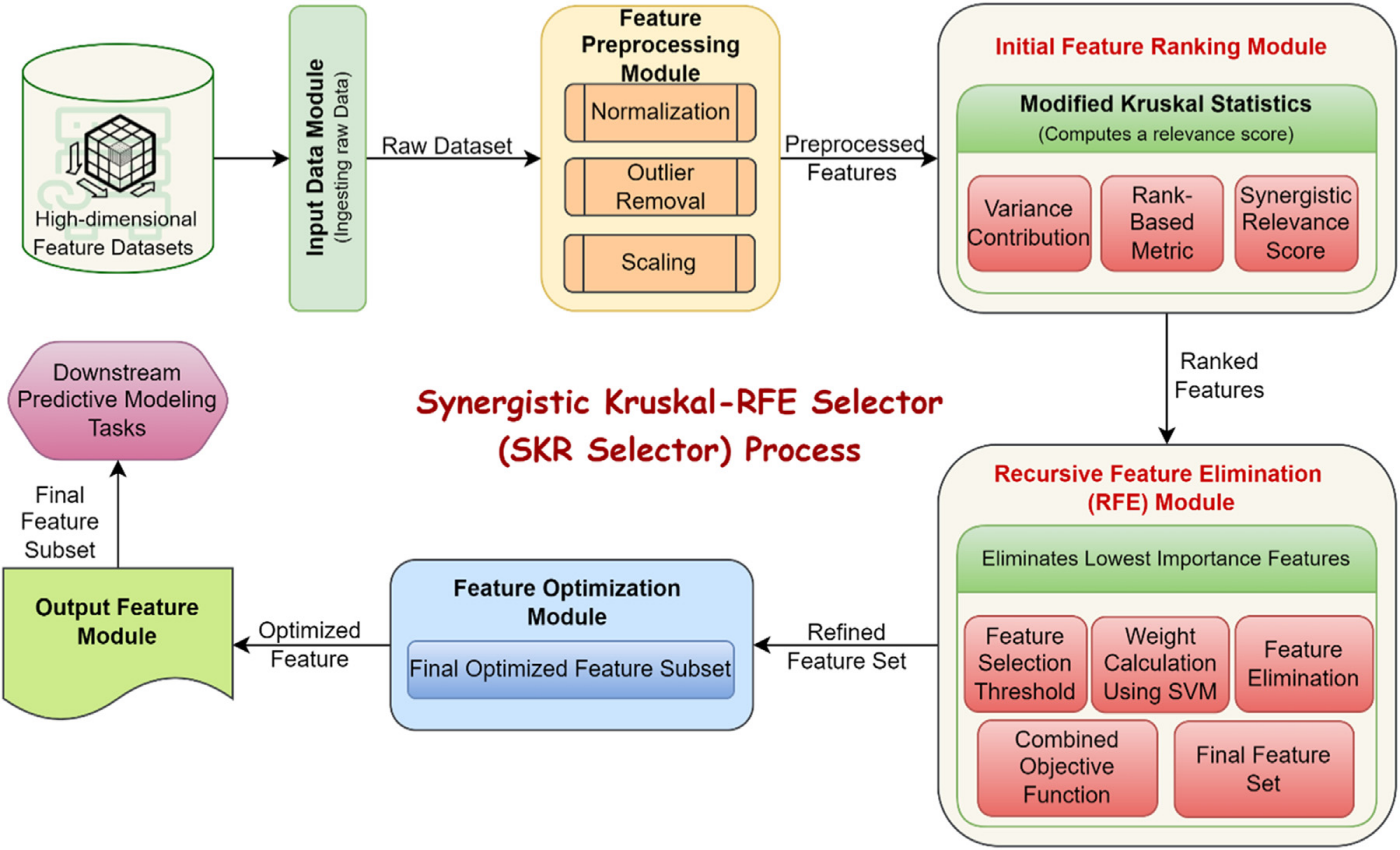
Synergistic Kruskal-RFE Selector (SKR Selector) Process.

The Synergistic Kruskal-RFE Selector (SKR Selector) is a novel method specifically developed to cope with restrictions of high dimensionality data space through the integration of feature ranking and sequence of elimination procedures. For this purpose, the SKR Selector employs a Kruskal-based mechanism simplified and married to the Recursive Feature Elimination (RFE) algorithm for selective feature ranking and optimal selection for classification, accuracy, and runtime. The concept of the algorithm is based on its capacity for integrating statistical information and subsequent enhancement for the selection of features relevant to predictive modeling [29]. The SKR Selector operates in two core stages: Two of the most important components of the SKR Selector are feature ranking and recursive elimination as shown in Fig. 2. In the first, variance and rank-based statistics are used to determine how useful each feature is in the ranking; in the second, the feature pool is gradually pruned based on classifiers’ weights. This two-step reductive process undertaken only once also helps to ensure that only the most promising features are passed on to downstream modeling stages which are bound to improve overall performance.

**Step 1: Initial Feature Ranking Using Modified Kruskal Statistics**

The first step in the SKR Selector is to compute a relevance score $S_{f_{i}}$ for each feature $f_{i}$ in the dataset D={X,Y}, where $X\in R^{N\times M}$ represents the feature matrix with N samples and M features, and $Y\in \{y_{1},y_{2},\ldots ,y_{k}\}$ denotes the target variable with kk unique classes. The relevance score is computed by combining two metrics: variance contribution and rank-based measures.

*Variance Contribution:* Variance quantifies the spread of a feature's values around its mean, providing insight into its potential importance in distinguishing between classes. For a feature $f_{i}$, the variance $V_{f_{i}}$ is calculated as:

(1) $$V_{f_{i}}=\frac{1}{N}\;\sum_{j=1}^{N}{\left(x_{ij}-\mu_{fi}\right)}^{2}$$

where $x_{ij}$ is the value of $f_{i}$ for the $j^{th}$ sample, and $\mu_{fi}$ is the mean of $f_{i}$, defined as:

(2) $$\mu_{f_{i}}=\frac{1}{N}\sum_{j=1}^{N}x_{ij}$$

A feature with higher variance may exhibit greater discriminatory power across classes. The variance contribution ensures that features with significant spread are prioritized in the ranking process.

*Rank-Based Metric:* To capture the relationship between feature values and class labels, a rank-based metric derived from Kruskal statistics is employed. This metric evaluates the separation of feature values among different classes by comparing their ranks. For a feature $f_{i}$, the rank-based metric $R_{f_{i}}$ is computed as:

(3) $$R_{f_{i}}=\sum_{c=1}^{k}\frac{n_{c}}{N}{\left({\bar{r}}_{c}-\bar{r}\right)}^{2}$$

Where $n_{c}$ is the number of samples in class c, ${\bar{r}}_{c}$ is the average rank of $R_{f_{i}}$ within class c, $\bar{r}\;$is the overall average rank of $f_{i}$ across all samples. Eqn. (3) ensures that features showing strong separability among classes receive higher rankings. The use of rank-based metrics reduces sensitivity to outliers, making it robust for diverse datasets.

*Synergistic Relevance Score:* The final relevance score $S_{f_{i}}$ for each feature $f_{i}$ is a weighted combination of variance contribution $V_{f_{i}}$ and rank-based metric $R_{f_{i}}$:

(4) $$S_{f_{i}}=\alpha \cdot V_{f_{i}}+\beta \cdot R_{f_{i}}$$

where α and β are parameters controlling the relative importance of variance and rank-based metrics. This synergy ensures that both statistical variability and class separation are considered in feature ranking.

**Step 2: Recursive Feature Elimination (RFE)**

The second stage of the SKR Selector is recursive feature elimination, which iteratively refines the feature set based on the rankings from Step 1. The process ensures that only the most relevant features are retained.

*Feature Selection Threshold:* At each iteration t, the algorithm retains a subset $X_{t}$ of the top $T_{t}$ features, where $T_{t}$ is determined as:

(5) $$T_{t}=T-\gamma \cdot t$$

with T being the total number of features and γ a predefined reduction factor controlling the rate of feature elimination.

*Weight Calculation Using SVM:* A Support Vector Machine (SVM) classifier is employed to assign importance weights $W_{f_{i}}$ to each feature. The weight $W_{f_{i}}$ for a feature $f_{i}\;$is computed as the magnitude of the corresponding component in the weight vector w:

(6) $$W_{f_{i}}=∥w∥2$$

where w is derived by solving the SVM optimization problem:

(7) $$\underset{w,b}{\underset{︸}{min}}\;\frac{1}{2}{∥w∥}_{2}^{2}+C\sum_{j=1}^{N}max\left(0,1-y_{j}\left(w^{⊤}x_{j}+b\right)\right)$$

with C being a regularization parameter and $y_{j}$ the label of the $j^{th}$ sample.

*Feature Elimination:* At each iteration, features with the smallest weights $W_{f_{i}}$ are eliminated. The process continues until the stopping criterion is met, such as a predefined number of iterations $t_{\text{max}}$ or a minimum feature count $T_{\text{min}}$.

*Combined Objective Function:* The SKR Selector optimizes a joint objective function that balances feature relevance, classification performance, and computational efficiency:

(8) $$L=\lambda_{1}\cdot \sum_{i=1}^{M}S_{f_{i}}+\lambda_{2}\cdot Acc\left(X_{T},Y\right)+\lambda_{3}\cdot \frac{1}{Time\left(T\right)}$$

Where, $Acc(X_{T},Y)$ is the classification accuracy achieved using the selected features $X_{T}$, Time(T) represents the computational time for evaluating T features, $\lambda_{1},\lambda_{2},\lambda_{3}$ are parameters controlling the trade-off between relevance, accuracy, and efficiency.

*Final Feature Set:* The output of the SKR Selector is the optimal feature subset $X_{opt}$ that maximizes the combined objective:

(9) $$X_{opt}=arg\underset{X_{T}}{\underset{︸}{max}}\;L$$

Eqn. (9) ensures that the selected features exhibit high relevance, contribute to better classification performance, and minimize computational costs. The SKR Selector provides a robust framework for feature selection in high-dimensional datasets. Its synergy between variance-based insights, rank-based metrics, and iterative refinement offers significant advantages in retaining only the most informative features, enhancing model performance in complex real-world scenarios.

Algorithm 1 Synergistic Kruskal-RFE Selector (SKR) Selector.

**Table:** 

**Input**: Dataset D={X,Y}, where $X\in R^{N\times M}$ and $Y\in \{y_{1},y_{2},\ldots ,y_{k}\}$
Parameters: α, β, γ, $T_{\text{min}}$, $t_{\text{max}}$, $\lambda_{1},\;\lambda_{2},\;\lambda_{3}$.
**Output**: Optimized feature subset $X_{opt}$.
**STEPS**
**1. Initialization:**
• Compute the initial number of features T=M. • Initialize relevance scores $S_{f_{i}}=0$ for each feature $f_{i}$, i=1,2,…,M. • Set the iteration counter t=0.
***Perform dataset-specific preprocessing:***
• *Numeric datasets:* Normalize or standardize features. • *Categorical datasets:* Apply encoding techniques. • *Signal data:* Extract signal-specific attributes. • *High-dimensional data:* Apply noise reduction techniques.
**2. Begin:**
**Step 1: Initial Feature Ranking Using Modified Kruskal Statistics**
1. **Compute Variance Contribution:**
○ **For** each feature $f_{i}$,
Compute variance $V_{f_{i}}$ by utilizing Eqn. (1).
2. **Compute Rank-Based Metric:**
○ **For** each feature $f_{i}$,
Compute rank-based metric $R_{f_{i}}$ by utilizing Eqn. (3).
3. **Compute Synergistic Relevance Score:**
○ Combine $V_{f_{i}}$ and $R_{f_{i}}$
Compute $S_{f_{i}}=\alpha \cdot V_{f_{i}}+\beta \cdot R_{f_{i}}$ by utilizing Eqn. (4).
**Step 2: Recursive Feature Elimination (RFE)**
1. **Initialize Feature Subset:**
○ Retain all T features in the subset $X_{t}$.
2. **Iterative Refinement:**
**While**$t<t_{\text{max}}\;$and $T>T_{\text{min}}$:
a. **Compute Feature Weights:**
○ Train an SVM classifier on $X_{t}$ and compute $W_{f_{i}}$ for each feature by utilizing Eqn. (6).
b. **Feature Elimination:**
○ Identify and remove features with the smallest $W_{f_{i}}$. ○ Update $T_{t}$ by utilizing Eqn. (5).
c. **Increment Counter::**
○ Increment t=t+1.
**Step 3: Dataset-Specific Customization**
1. **Numeric Datasets:**
○ Focus on variance and relevance for numeric features.
○ Use Kruskal Variance Ranking (KVR) for feature ranking.
2. **Categorical Datasets:**
○ Encode and integrate categorical features using rank-based metrics that account for class separability.
3. **Signal Data:**
○ Adapt feature extraction to signal-specific attributes like frequency and amplitude.
○ Combine these attributes with the standard ranking mechanism.
4. **High-Dimensional Data:**
○ Apply noise reduction techniques to improve relevance and efficiency.
○ Enhance RFE by prioritizing biologically significant features.
**Step 4: Optimization and Selection:**
**1. Combined Objective Function:**
○ Evaluate the joint objective function by utilizing Eqn. (8).
**2. Final Feature Set:**
○ Select the optimal feature subset $X_{opt}$ that maximizes L by utilizing Eqn. (9).
**3. Return:**
• Return the optimal feature subset $X_{opt}$.

#### Distributed multi-kernel classification framework (DMKCF)

**Fig. 3:**
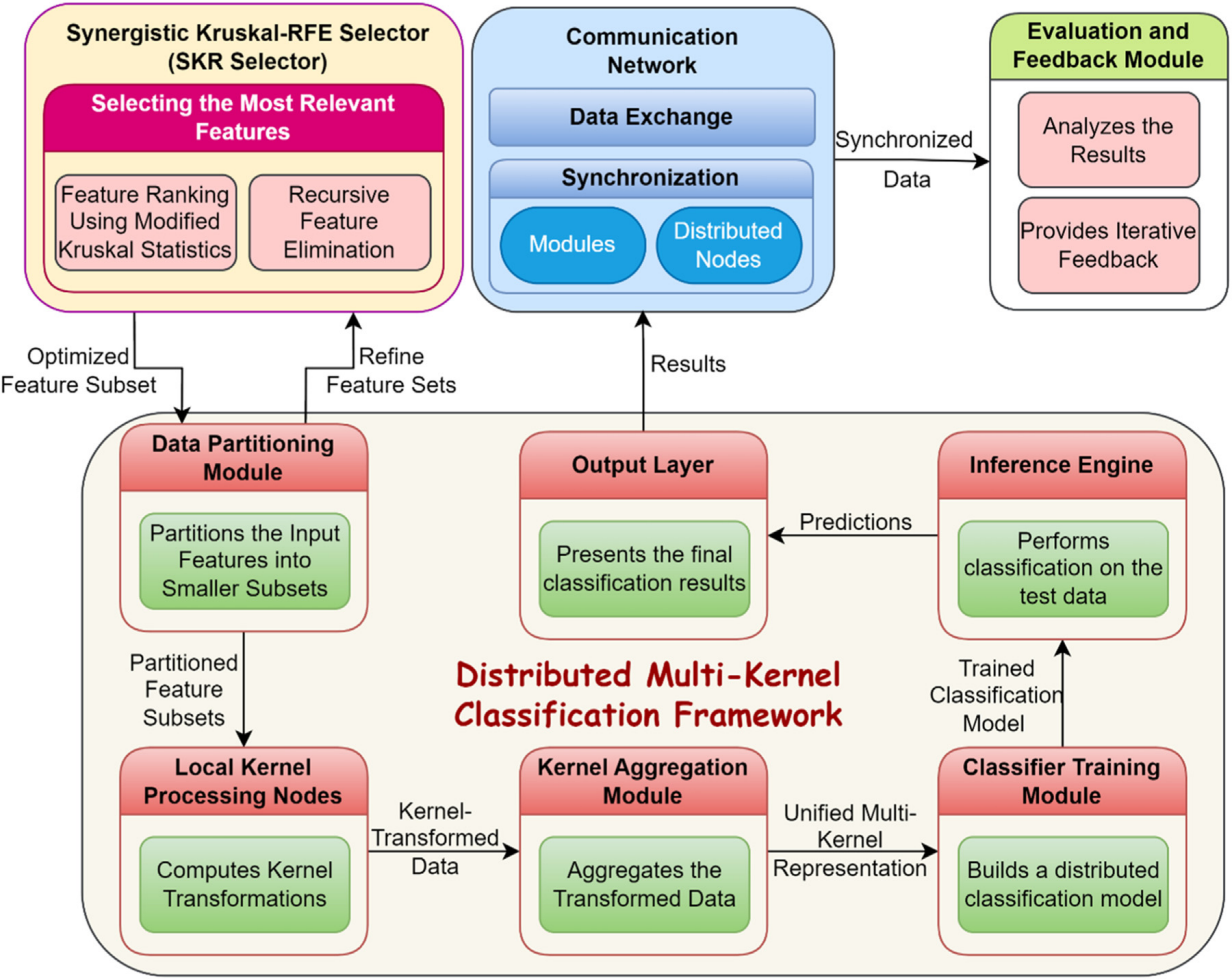
Distributed Multi-Kernel Classification Framework (DMKCF).

The DMKCF is a versatile methodology addressing the need for integrating multi-kernel learning and embedded distributed characteristics. The elements of this framework were developed to offset the computational and scalability issues of analyzing massive datasets with many features in real-time distributed systems. Through the use of multi-kernel learning, DMKCF can easily deal with different kinds of data and still maintain scalability since the tasks can be partitioned in a parallel manner among the nodes as shown in Fig. 3. Kernel-based methods play a central role in machine learning because they transform the originally non-separable data space into a higher-dimensional space, for which linear classification is possible. For a given kernel function $k(x,\;x^{\prime })$, the transformation is expressed mathematically as:

(10) $$k\left(x,\;x^{\prime }\right)=\;\phi {\left(x\right)}^{⊤}\phi \left(x^{\prime }\right)$$

where ϕ(x) is the mapping function that projects input data x into a high-dimensional space. Multi-kernel learning extends this principle by combining multiple kernel functions, each tailored to different data characteristics. The composite kernel in DMKCF is defined as:

(11) $$K\left(x,\;x^{\prime }\right)=\;\sum_{\left\{m=1\right\}}^{M}\beta_{m}k_{m}\left(x,\;x^{\prime }\right)$$

Where, M is the total number of kernels, $k_{m}\left(x,\;x^{\prime }\right)$ represents the m-th kernel function, $\beta_{m}$ is the weight assigned to each kernel, subject to constraints $\beta_{m}\geq 0$ and $\sum_{\left\{m=1\right\}}^{M}\beta_{m}=1$. Eqn. (11) highlights how the composite kernel integrates diverse perspectives, capturing both linear and non-linear relationships. The optimization process in DMKCF focuses on learning the optimal weights $\beta_{m}$, ensuring adaptability across varying data distributions. Multi-kernel learning is advantageous as it simultaneously leverages the strengths of multiple kernels, avoiding the limitations of single-kernel methods, which may underperform on complex datasets. Table 1 presents a summary of all symbols and definitions used in the proposed work.

**Table 1:** Notation and Meanings.

Notation	Explanation
$f_{i}$	Feature
$V_{f_{i}}$	Variance
$\mu_{fi}$	Mean of $f_{i}$
$R_{f_{i}}$	Rank-Based Metric
$S_{f_{i}}$	Relevance Score
${\bar{r}}_{c}$	Average Rank of $R_{f_{i}}$
$\bar{r}$	Overall Average Rank of $f_{i}$
$W_{f_{i}}$	Weight for a Feature $f_{i}$
$Acc(X_{T},Y)$	Classification Accuracy
D={X,Y}	Dataset
α, β, γ	Parameters
$\lambda_{1},\;\lambda_{2},\;\lambda_{3}$	Parameters
$X_{opt}$	Optimal Feature Subset
$k(x,\;x^{\prime })$	Kernel Function
$\beta_{m}$	Weight Assigned to Each Kernel
ϕ(x)	Mapping Function
$D_{j}$	Subset
$l(y_{i},\;f\left(x_{i}\right))$	Loss Function
C	Regularization Parameter

**Distributed Architecture for DMKCF**

The distributed architecture of DMKCF is pivotal in handling massive datasets by dividing computations among multiple nodes in a network. Let the dataset $D\;=\;{\left\{\left(x_{i},\;y_{i}\right)\right\}}_{i=1}^{N}$ consist of N samples, where $x_{i}\in \;R^{d}$ represents input data, and $y_{i}\;\in \;\left\{-1,\;+1\right\}$ denotes the corresponding labels. This dataset is partitioned across P nodes, with each node processing a subset $D_{j}$. At each node, the local kernel matrix is computed as:

(12) $$K_{j}\left(x_{i},\;x_{i}^{\prime }\right)=\;\sum_{\left\{m=1\right\}}^{M}\beta_{m}k_{m}\left(x_{i},\;x_{i}^{\prime }\right),\;\forall x_{i},\;x_{i}^{\prime }\in D_{j}$$

Where j∈{1,2,…,P} represents the node index. These local computations are aggregated to form the global kernel matrix:

(13) $$K\;=\;\sum_{\left\{j=1\right\}}^{P}K_{j}$$

Eqs. (12) and (13) illustrate how DMKCF achieves scalability by parallelizing the kernel computations while maintaining the coherence of the global optimization process. This distributed strategy is essential for reducing latency and ensuring that the framework can scale to applications with high computational demands, such as real-time decision-making systems or large-scale image classification tasks. The primary objective of DMKCF is to derive a decision function f(x) that minimizes classification error while maintaining model complexity. The decision function is formulated as:

(14) $$f\left(x\right)=\;\sum_{i=1}^{N}\alpha_{i}K\left(x,\;x_{i}\right)+\;b$$

Where $\alpha_{i}\;$are the dual coefficients, b is the bias term. The classification problem is cast into a regularized optimization problem:

(15) $$\underset{\left\{\alpha ,\;b\right\}}{\underset{︸}{min}}\frac{1}{2}\sum_{\left\{i,\;j\right\}}\alpha_{i}\alpha_{j}K\left(x_{i},\;x_{j}\right)+\;C\sum_{\left\{i=1\right\}}^{N}\ell \left(y_{i},\;f\left(x_{i}\right)\right)\;$$

Eqn. (15) subject to the constraints $\sum_{\left\{i=1\right\}}^{N}\alpha_{i}y_{i}=0$ and $\alpha_{i}\geq 0$. In Eq. (15): $l(y_{i},\;f\left(x_{i}\right))$ represents the loss function, typically the hinge loss $\max(0,1-y_{i},\;f\left(x_{i}\right)),\;C$ is a regularization parameter that balances model complexity and empirical risk minimization. Eqn. (15) encapsulates the trade-off between ensuring that the model generalizes well to unseen data while fitting the training data accurately. The optimization in DMKCF is performed in a distributed manner using the Alternating Direction Method of Multipliers (ADMM). The global optimization problem is decomposed into sub-problems that are solved locally at each node. The local objective at node j is:

(16) $$\left\{L_{j}\left(\alpha_{j},\;z\right)\right\}=\frac{1}{2}{\left|\left|\alpha_{j}\right|\right|}^{2}+\;C\;\sum_{i\;\in {\left\{D\right\}}_{j}}l\left(y_{i},\;f\left(x_{i}\right)\right)+\;\rho {\left|\left|z\;-\alpha_{j}\;\right|\right|}^{2}$$

where z is the global variable synchronized across nodes, ρ is the penalty parameter controlling the trade-off between local and global coherence. Eq. (16) ensures that the local optimization aligns with the global objective, leveraging distributed resources effectively.

**Advantages of DMKCF**

The DMKCF framework introduces several advantages, making it a robust choice for large-scale classification problems:

*Scalability:* By distributing computations, DMKCF can efficiently process massive datasets without compromising on speed or accuracy.

*Flexibility:* The multi-kernel approach enables the integration of diverse data representations, capturing intricate patterns that single-kernel methods might miss.

*Robust Optimization:* The use of regularized loss functions and distributed optimization ensures that the framework adapts well to dynamic data distributions while maintaining computational efficiency.

*High Performance:* The combined strength of kernel learning and distributed processing allows DMKCF to deliver state-of-the-art classification results across various domains, from image recognition to real-time data analytics.

Algorithm 2 Distributed Multi-Kernel Classification Framework (DMKCF).

**Table:** 

**Input**:
• Dataset D distributed across P nodes as $D_{j}$.
• Parameters C, ρ, ϵ.
• Kernel functions $k_{m}\left(x,x^{\prime }\right)$, m∈{1,2,…,M}
**Output**:
• Decision function f(x).
**Steps**
**1. Initialize:**
• ***Data Partitioning****:* Partition D across P nodes into subsets $D_{j}$
• ***Initialize*** kernel weights $\beta_{m}=\frac{1}{M}$, dual coefficients $\alpha_{j}=0$, and global synchronization variable z=0.
• ***Set*** the iteration counter t=0.
**2. Compute Local Kernel Matrices:**
• ***For*** each node j,
***Compute*** the local kernel matrix by utilizing Eqn. (12).
• ***Aggregate*** the local kernel matrices to form the global kernel matrix, by utilizing Eqn. (13).
**3. Iterative Optimization:**
**(a) Local Update at Each Node**j**:**
• Solve the local optimization problem described by Eqn. (16).
• Update the dual coefficients $\alpha_{j}$ based on the local dataset $D_{j}$ and z.
**(b) Global Synchronization:**
• Synchronize the global variable z by averaging $\alpha_{j}$ values across all nodes: $z=\;\frac{1}{P}\sum_{j=1}^{P}\alpha_{j}$.
**(c) Kernel Weight Update:**
• Adjust the kernel weights $\beta_{m}$ dynamically to optimize the composite kernel, by utilizing Eqn. (11).
**(d) Convergence Check:**
• **If** the difference $∥z^{t+1}-z^{t}∥\;<\epsilon$ (using the convergence criterion):
○ Exit the loop.
• **Else:**
○ Increment t and repeat.
**4. Construct Decision Function:**
• After convergence, construct the decision function f(x) using the kernel matrix and coefficients, by utilizing Eqn. (14).
**5. Return:**
• Return the optimal decision function by utilizing f(x).

## Method validation

### Results and discussion

The proposed Synergistic Kruskal-RFE Selector (SKR Selector) and Distributed Multi-Kernel Classification Framework (DMKCF) were tested and compared in order to understand their capability and efficacy in feature selection and classification. This section reports the results of the above-mentioned experiments in terms of comparison with the alternatives as well as a discussion on the implications of these experiments. The performance of the SKR Selector was revealed to extract the most suitable features from the high-dimensional data. As the accuracy of other classification tasks that followed the feature selection process was either preserved or enhanced, the SKR Selector integrated Kruskal's Minimum Spanning Tree algorithm and Recursive Feature Elimination (RFE) in parallel. This process not only reduced computational cost but also helped the model interpretability which is an important feat to consider in any data-driven application where understanding features impacts is important. Based on the selected features, the DMKCF tackled the issues with large-scale data classification by using a distributed multi-kernel scheme. The DMKCF was able to maintain sufficient classification accuracy derived from the aggregation of the outputs of different kernel types on various datasets. It had a distributed form of processing that executed tasks in parallel, hence scalability and in general, less computational time. The expansion of the kernel space provided the capability to apply the framework to various kinds of data structures and indeed provided a marked enhancement of the classification accuracy over the traditional single kernel modes. In addition to the benchmark datasets, real applications were used to test the performance of the proposed approach. Evaluation performance measures which include accuracy, precision, recall, specificity, and computer time were used in the research. The results obtained revealed that the proposed methods, namely the SKR Selector and DMKCF achieved better results and lower computational complexity than common methods, especially for high-dimensional databases.

#### System configurations

Table 2 provides a detailed description of system configurations provides a solid foundation for replicating the experiments and understanding the performance enhancements achieved by our methods. The proposed methods were started and executed in a clear computing environment so that maximum efficiency and replicability could be obtained. To build the tool, Python (Version 3.8) was used, along with the following libraries: Scikit-learn for the RFE process and NetworkX for mapping and computation of Kruskal's Minimum Spanning Trees. The hardware used for the SKR Selector comprised of a processor Intel Core i5–9600 K @ 3.7 GHz and memory of RAM 16 GB. This implementation was done on a locally installed system operating on Ubuntu 20.04 LTS. The DMKCF employed a distributed computing environment built with Apache Spark (Version 3.0.1) with a Java backend. This framework used Spark MLlib for the kernel's efficiency on operations and the inclusion of very diverse multi-kernel aggregation scripts. Cluster topography was set with one master node containing the Intel Xeon CPU E5–2670 @ 2.6 GHz and 32 GB RAM and four worker nodes containing the Intel Xeon E5–2670 @ 2.6 GHz and 16 GB RAM each. These nodes were connected through a high-speed, Wide Area Network with a bandwidth of 1Gbps. The distributed computations were done on Hadoop Distributed File System abbreviated as HDFS, to effectively process data over the cluster. The operating system to support this environment was CentOS 7 which is stable and compatible with the distributed framework. This consistently strong system configuration allowed the proposed methods to proceed optimally and at large scale and high dimensionality, thus both addressing and resolving computational difficulties related to big data and large dimensions.

**Table 2:** System Attributes and Configurations.

Attribute	Value
Programming Language	Python (SKR Selector), Java (DMKCF)
Framework	Scikit-learn, NetworkX (SKR Selector), Apache Spark (DMKCF)
Feature Selection Library	Scikit-learn RFE module
Kernel Processing	Spark MLlib
Master Node	Intel Xeon CPU E5–2670 @ 2.6 GHz, 32 GB RAM
Worker Nodes	4 × Intel Xeon E5–2670 @ 2.6 GHz, 16 GB RAM
Network Bandwidth	1 Gbps
Operating System	Ubuntu 20.04 LTS (SKR Selector), CentOS 7 (DMKCF)
Dataset Storage	HDFS for distributed kernel computations

#### Dataset

The proposed approaches were tested on the four different datasets from medical and biological domains to compare the performance and effectiveness of the proposed methods and investigate their properties. PIMA Indian Dataset is applied mostly for diabetes prediction; it contains medical attributes like glucose level, blood pressure, BMI, and age of the patients that make it reasonable for experiments with numeric health-related predictors testing. The Kaggle Heart Disease Dataset contains a set of features related to cardiovascular health, such as cholesterol level, age, gender, and ECG, to make it possible to assess the classification framework for handling the numerical as well as categorical data. For neurological health, the Kaggle Parkinson's Disease Dataset aims to detect Parkinson's disease through vocal measurements such as frequency and amplitude components of sustained phonations that show the applicability of the framework in analyzing non-conventional biomedical signals. Lastly, Kaggle Multi-Omics TCGA Data based on the TCGA project provides high-dimensional data containing genomic, transcriptomic, epigenomic, and proteomic information for multi-omics integration and cancer prediction, which is a challenging benchmark dataset. Combined, these datasets exemplify the effectiveness and feasibility of the proposed SKR Selector and DMKCF for various real-world, high-dimensional classification applications.

#### Feature selection across diverse datasets

A customized approach was evaluated for each of the four datasets that were used to evaluate the proposed SKR Selector, each of which required different feature types from several diverse domains. Since the PIMA Indian Diabetes Dataset mostly contains numeric features, the SKR Selector is used to rank the feature variance and relevance to diabetes prediction using Kruskal Based Variance Ranking (KVR), then remove redundant features with Recursive Feature Elimination (RFE). The Kaggle heart disease dataset which contains a numeric and categorical feature such as cholesterol level and ECG readings. The diversity is managed in the SKR Selector by an encoding technique and a ranking mechanism that takes into account both data types to choose relevant predictors. The non-conventional biomedical signal is represented by Kaggle Parkinson's Disease dataset which presents vocal measurements such as frequency and amplitude. In this paper, we employ the SKR Selector to adapt to the signal-specific attributes with a combined approach of KVR and RFE to find the most discriminative vocal patterns. The challenges in the final domain are finally posed by the complex, high-dimensional Kaggle Multi-Omics TCGA Dataset consisting of genomic, transcriptomic and epigenomic data. This problem is addressed in the SKR Selector which takes a more noise reduction and dimensionality reduction approach by keeping only the most biologically relevant features. Finally, this tailored feature selection process highlights how robust and flexible the SKR Selector is, for which it can effectively perform classification and analysis in variety of biomedical datasets, thus improving the framework's generalizability and applicability.

#### Performance evaluation

Metrics for performance evaluation consist of an extensive list in order to evaluate the effectiveness of the presented methodologies. Measures of classification including accuracy, precision, specificity, and Recall were employed in making an assessment of the ability of the frameworks to classify data instances with efficiency and effectiveness across different datasets. When analyzing the performance of feature selection, the Feature Reduction Ratio which denotes the ratio of the number of features selected to the number of all the features, as well as the Relevance Score, which estimates the relevance of features were used. In addition, computational efficiency metrics such as computational time, speedup time, and memory consumption were considered in order to compare the performance and scalabilities of the two proposed frameworks. The outcome of these evaluations was compared with conventional feature selection algorithms, such as Recursive Feature Elimination (RFE), Random Forest Feature Selection Technique (RFS), Recursive Feature Elimination with Cross-Validation (RFECV), and Support Vector Machine – Recursive Feature Elimination (SVM-RFE) to demonstrate the existing novel techniques and the proposed approaches’ strengths.

##### Classification performance metrics

The classification performance of the proposed method is measured normatively, usually in terms of accuracy, precision, specificity, and recall. Accuracy displays the number of appropriately predicted instances to the ratio of all the samples and offers the general assessment of the model's predictiveness. Precision is the measure of the proportion of instances that were correctly classified as positive out of all instances classified as Positive, which is very vital in preventing large numbers of false positives. Specificity measures how accurately the model is in detecting the presence of negative cases, and here, few false alarms would be revealed. Specificity refers to the ability to avoid the detection of non-cases in applications with an emphasis on minimizing false positives because Recall (also called sensitivity) quantifies the proportion of actual positives that are identified. These overall measures collectively form a ubiquitous assessment matrix that points out the degree of strengths and weaknesses of the models being considered. The feature selection and classification algorithms like the SVM-RFE, RFECV, RFS, RFE, and the proposed SKR-DMKCF are compared in terms of sensitivity, specificity, accuracy, and the Matthews correlation coefficient across the different datasets which are used to ensure relativism and robustness of the results obtained.

Table 3 shows the comparison with the baseline methods SVM-RFE, RFECV, RFS, and RFE the proposed SKR-DMKCF framework achieves consistently better classification performance on multiple datasets. Accuracy, Precision, Specificity, and Recall all emphasize different aspects of the proposed method and important enhancements are demonstrated in different situations. Regarding accuracy, the proposed SKR-DMKCF achieves greater improvement compared to all the other methods in different datasets. For instance, in Dataset 1, its accuracy is 87 %, which is 4.8 % more than the second-best method, RFECV with 83 %. This enhancement raises the bar of the effectiveness of the optimization in SKR-DMKCF by incorporating improved feature selection and classification mechanisms for accurate recognition of such features. Similarly in dataset 4, SKR-DMKCF achieves an 89 % Accuracy which is 2.3 % more than in RFECV (87 %). The precision values also emphasize hyperparameters' decision-making significance regarding to exclusion of false positives pertaining to SKR-DMKCF. Similarly, the precision of enhancing the externally validated gene sets from Dataset 3 is 80 % for SKR-DMKCF while the RFECV is 73 % which is 7 % higher. This proves that the proposed method, SKR-DMKCF, can filter out the least important features, thereby maximizing the reliability of the classification results. In cases of more difficult class distributions of Dataset 2, precision is 79 % while for RFECV, it is 6 % less than that of the SKR-DMKCF model.

**Table 3:** Classification Performance Comparison.

Datasets	Methods	Accuracy	Precision	Specificity	Recall
Dataset 1	SVM-RFE	78	75	82	76
	RFECV	83	79	86	80
	RFS	73	69	77	70
	RFE	69	65	73	66
	SKR-DMKCF	87	83	90	86
Dataset 2	SVM-RFE	74	69	78	72
	RFECV	79	73	82	77
	RFS	67	64	72	66
	RFE	64	60	68	63
	SKR-DMKCF	82	79	88	81
Dataset 3	SVM-RFE	71	70	79	73
	RFECV	72	73	82	78
	RFS	64	65	74	67
	RFE	60	61	70	63
	SKR-DMKCF	83	80	89	85
Dataset 4	SVM-RFE	82	78	84	77
	RFECV	87	83	89	82
	RFS	76	73	78	71
	RFE	71	70	74	68
	SKR-DMKCF	89	84	91	88

**Fig. 4:**
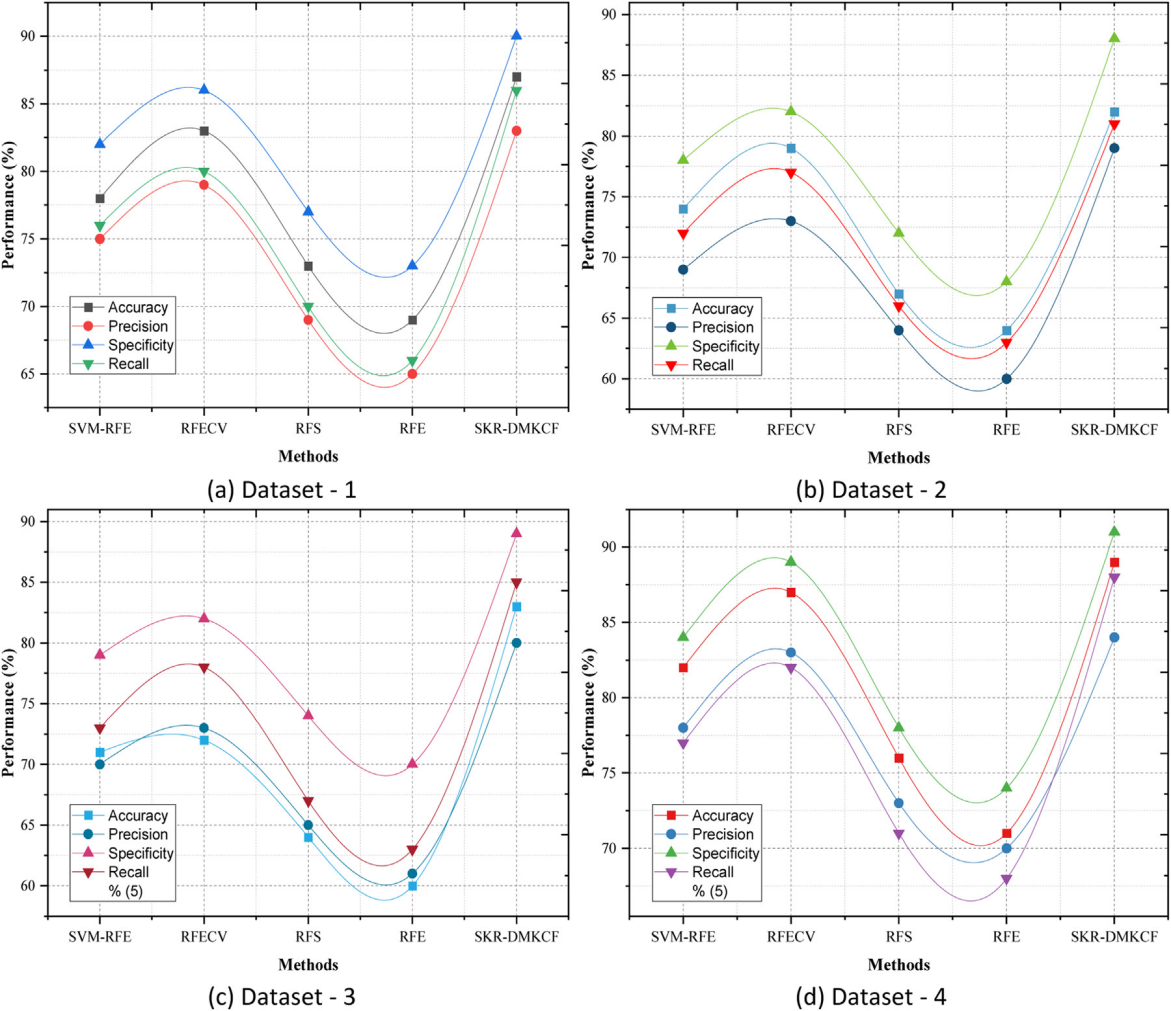
Classification Performance Comparison.

When evaluating specificity, which focuses on the true negative rate, the performance of the proposed SKR-DMKCF method is quite stable. For example, in Dataset 2, the proposed method SKR-DMKCF gets 88 % of the specificities while RFECV only gets about 82 % of specifics as shown in Fig. 4. This improvement is necessary for the application specialization at which high accuracy may be needed in labeling non-relevant or negative samples to avoid any misclassification. In the same vein, SKR-DMKCF achieves 90 % of the specificity, which is 4 % higher than the RFECV method confirming the possibility of appropriately filtering out negative patterns. Lastly, recall, following the framework's emphasis on identifying relevant samples, also demonstrates SKR-DMKCF's stability again. In Dataset 4, SKR-DMKCF obtained 88 % recall, while RFECV obtained 82 % recall only, and thereby improved by 6 %. The recall values obtained for Dataset 1 were also not different from the trends observed for accuracy; SKR-DMKCF got a recall of 86 %, which is 6 % higher than that of RFECV, with a recall of 80 %. This improvement is important for applications where all possible samples must be identified and distinguished to avoid fatal misclassifications. From Table 3 it can be seen that not only SKR-DMKCF yields higher accuracy than the current methods, but it also enhances the performances of accuracy, precision, specificity, and recall in a range of 4 %−7 % in all the tested scenarios. These results confirm the performance of the SKR-DMKCF framework in terms of feature selection and classification.

##### Feature selection effectiveness metrics

To assess the efficiency and impact of the feature selection process, two key metrics were utilized: The feature reduction ratio and the relevance score of the selected features were also presented as figures. In combination, all of them ensure the adequate evaluation of the feature selection process, selecting features not only decreases the data dimensionality but also increases the relevance and quality of the input for further classification. These metrics are further discussed in Table 4, showing a better performance of the proposed method in comparison with the conventional method.

**Table 4:** Comparison of Feature Reduction Ratio.

Datasets	SVM-RFE	RFECV	RFS	RFE	SKR-DMKCF
**Dataset 1**	0.82	0.85	0.78	0.76	0.89
**Dataset 2**	0.84	0.87	0.79	0.76	0.91
**Dataset 3**	0.81	0.84	0.77	0.73	0.87
**Dataset 4**	0.83	0.86	0.79	0.76	0.9

***Feature Reduction Ratio***

The **Feature Reduction Ratio** measures the proportion of features retained after the selection process compared to the original set of features. This metric provides insight into the algorithm's capability to reduce the dimensionality of data, which is essential for minimizing computational overhead and enhancing model interpretability. Mathematically, it is defined as:

(17) $$Feature\;Reduction\;Ratio\;\left(FRR\right)=\frac{Number\;of\;Selected\;Features}{Total\;Original\;Features}\times \;100$$

**Fig. 5:**
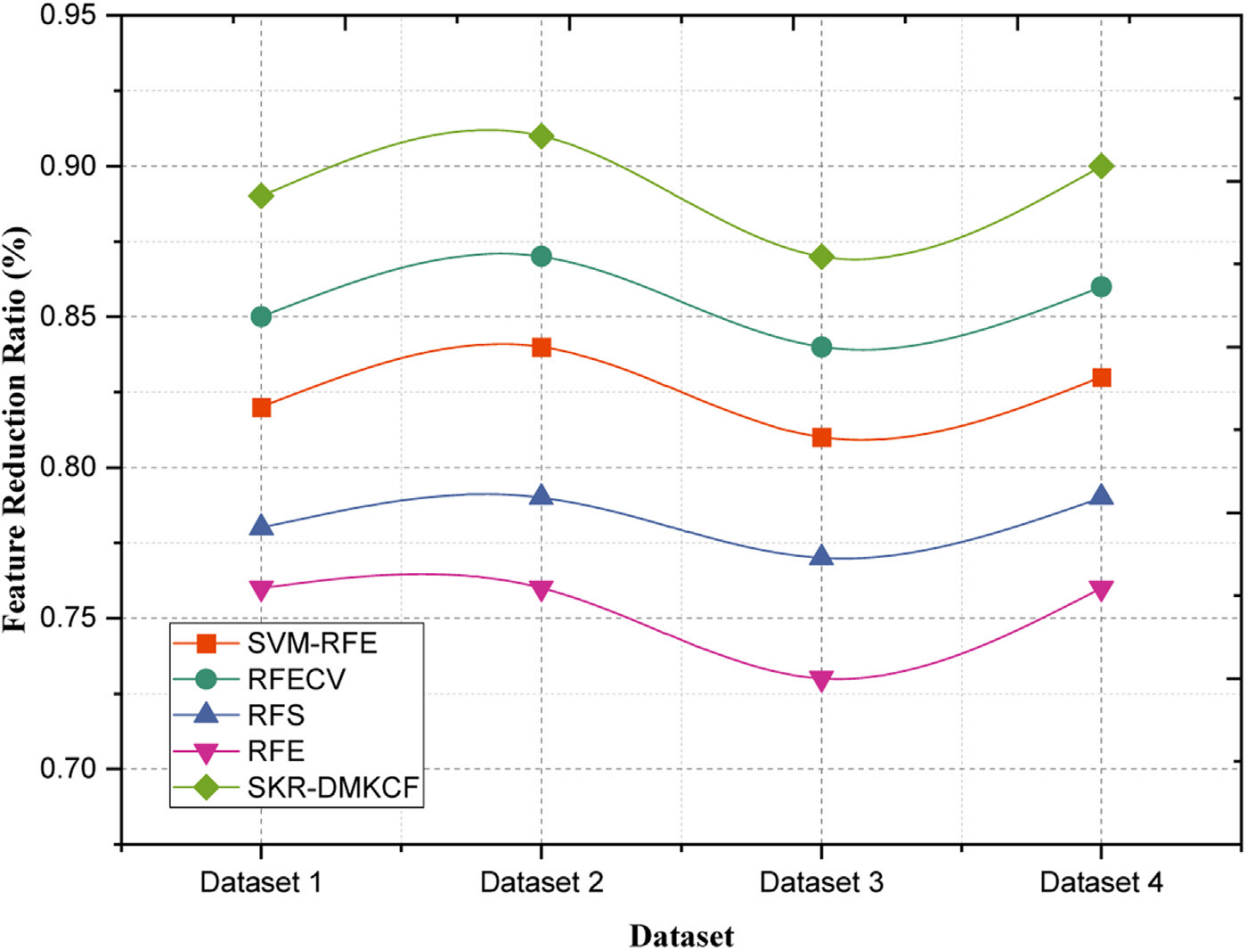
Comparison of Feature Reduction Ratio.

The Feature Reduction Ratio table gives the comparison of the various methods used in feature selection in an ability to reduce the dimensions of the datasets. That way, the ratio is even higher, which means that the method can preserve crucial aspects and use the least number of features in total. The Feature Reduction Ratio of SKR-DMKCF for Dataset 1 is 0.89, remaining 4.7 % better compared to the second-best method, RFECV with 0.85. The computational results illustrate that the SKR-DMKCF algorithm is faster than RFE (0.76) with an improvement of 17.1 % which establishes the effectiveness of its dimensionality reduction algorithm without losing equally crucial features. A relatively high efficiency of 0.91 is recorded by applying SKR-DMKCF in Dataset 2; this makes a 4.6 % improvement compared to RFECV (0.87) and an 8.3 % improvement over SVM-RFE (0.84) as shown in Fig. 5. Specifically, in the context of RFE (0.75), SKR-DMKCF boasts a 19. 7 % difference strengthening the argument that it is integrated with a rigid filter model which screens of most of the features.

For Dataset 3 the ratio of SKR-DMKCF is 0.87 which is 3.6 % than RFECV (0.84) and 7.4 % than SVM-RFE (0.81). Compared to RFE we found that the proposed method has improved by 19.2 % than RFE (0.73) thus proving its efficiency and effectiveness in feature selection for better computational time and accuracy. In the fourth dataset, the highest ratio of 0.90 is obtained by the proposed SKR-DMKCF, and this is 4.7 % better than RFECV (0.86) and 8.4 % better than SVM-RFE (0.83). Comparing it with RFE which gave 0.76, SKR-DMKCF shows an additional 18.4 % of feature selection effectiveness in terms of ability to manage dimensionality reduction as well as feature retention. These results unambiguously show that SKR-DMKCF achieves a higher Feature Reduction Ratio across the datasets than the other models and, therefore, can be recommended for reducing the computational load while preserving informative features.

***Relevance Score***

The Relevance Score is a ratio that shows the relevance of the selected features to the predictive performance of the model. This is arrived at by calculating the weights or importance given to each feature while selecting features for analysis; thereby underlining the bandwidth of the algorithm in giving high importance to features that are important for proper classification. The Relevance Score is determined as the level of impact or indispensability of the chosen variables to the model's accuracy. It can be expressed mathematically as:

(18) $$Relevance\;Score\;\left(RS\right)=\frac{\sum_{i=1}^{n}Weight\left(f_{i}\right)\cdot Performance\;Gain\left(f_{i}\right)}{n}$$

Eqn. (18) evaluates the contribution of each selected feature to the overall performance of the model. In this context, $f_{i}$ represents the $i^{th}$ selected feature, which is part of the feature subset chosen by the feature selection algorithm. The term Weight ($f_{i}$) indicates the importance or weight assigned to the $i^{th}$ feature, reflecting its relative significance in the model. Meanwhile, Performance Gain ($f_{i}$) quantifies the improvement in performance metrics (such as accuracy, precision, or recall) directly attributable to the inclusion of the $i^{th}$ feature. Finally, n refers to the total number of selected features in the subset. This formula computes the average contribution of all selected features to the model's performance, emphasizing the effectiveness of the feature selection process.

**Fig. 6:**
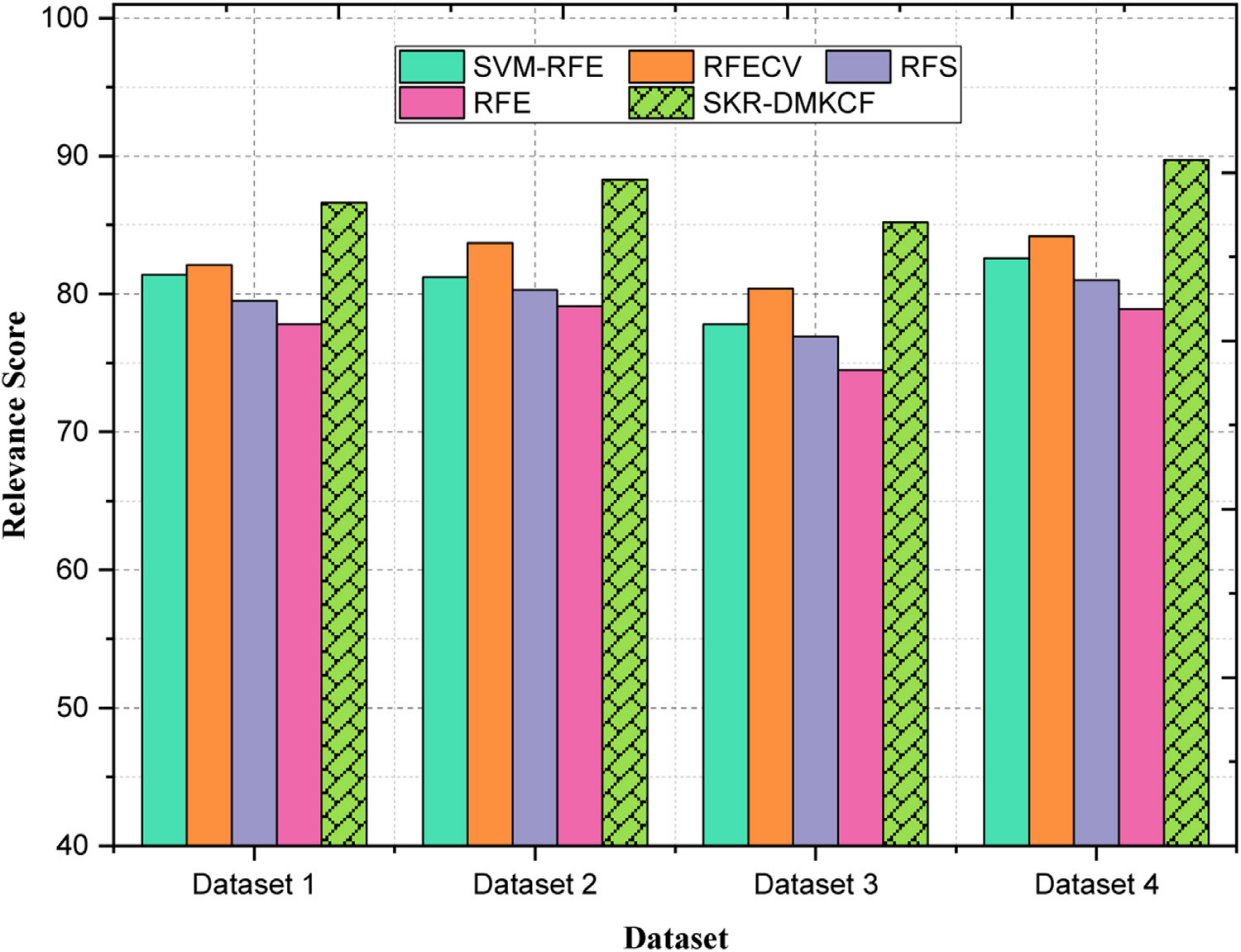
Comparison of Relevance Score (RS).

Table 5 shows a comparison of different feature selection methods as to how effectively they could rank the most important features for the datasets in question. Hence, the Relevance Score increases, which indicates the ability of the method to choose features that affect its performance most. Overall, for the first dataset, the highest Relevance Score 86.6 belongs to the SKR-DMKCF while the second best is RFECV scoring 82.1. The results of SKR-DMKCF have shown about 11.3 % better feature selection precision compared to that of RFE (77.8) as shown in Fig. 6. Similarly in Dataset 2, SKR-DMKCF achieved an RS of 88.3 which outperforms RFECV with 5.5 % and SVM-RFE with 8.8 %. By comparing the results obtained with RFE which yielded a 79.1 score, it is clear that in SKR-DMKCF mode, 11.7 % improvement was realized, and the understanding of the efficiency of the proposed modes to work always in the direction of considering relevant features. With regards to Dataset 3, the best results for SKR-DMKCF have an RS of 85.2 which is 6.00 % higher than RFECV 80.4 and 9.50 % higher than SVM-RFE with 77.8 score. Compared with RFE (74.5), the present method of SKR-DMKCF is higher by 14.3 %, which again supports the better performance of the SKR-DMKCF in feature selection. In Dataset 4, by achieving an overall detection rate of 89.7, SKR-DMKCF has better performance than all the other algorithms with an RS of 6.5 % compared to RFECV with 84.2 and 8.6 % compared to SVM-RFE with 82.6. It is higher than RFE which is 78.9 in enhancing the performance of the model through improving the features selection by 13.7 %. These results effectively help to highlight the usefulness of the SKR-DMKCF approach as a superior performer in terms of the Relevance Scores for all the tested datasets in different experiments, further asserting SKR-DMKCF's capacity to reveal notable features and rank them accordingly. Ongoing performance indicates its relevance, particularly in deciding on the feature selection process when it enhances classification results.

**Table 5:** Comparison of Relevance Score (RS).

Datasets	SVM-RFE	RFECV	RFS	RFE	SKR-DMKCF
**Dataset 1**	81.4	82.1	79.5	77.8	86.6
**Dataset 2**	81.2	83.7	80.3	79.1	88.3
**Dataset 3**	77.8	80.4	76.9	74.5	85.2
**Dataset 4**	82.6	84.2	81.0	78.9	89.7

##### Computational efficiency metrics

Computational Efficiency Metrics are essential in determining the scalability and the practicality of the algorithm in a resource-constrained environment context. Such metrics include computational time which is the time taken by the algorithm to execute; speed-up time which quantifies the amount of time it took to execute when optimization techniques or parallel processing has been employed; and memory used which measures the amount of memory used during the process of operation of the algorithm. Combined, these figures give a clear picture of the resources that the algorithm consumes and can be used in comparing different methods and selecting the optimal method in terms of computational load. The assessment of these measures not only represents the performance of the advanced SKR-DMKCF framework but additionally proves the superiority of the framework in terms of time and resources over conventional approaches.

***Computational time***

Computational Time is a critical measure used to evaluate the efficiency of an algorithm, particularly in scenarios requiring rapid processing of input data. It quantifies the total time consumed from the moment input is received until the desired output is generated. This metric is essential for determining the practicality of an approach, especially for real-time or resource-constrained environments. A formula for calculating Computational Time (CT) is expressed as:

(19) $$CT\;=\;\sum_{j=1}^{m}N_{j}\cdot T\left[q_{j}\right]$$

In Eqn (19), CT represents the cumulative computational time, measured in milliseconds (ms). $N_{j}$ refers to the count of specific tasks or input cases processed during the $j^{th}$ computation cycle. $T[q_{j}]$ denotes the time taken to process the $j^{th}$ task or input case, with m representing the total number of tasks or computation cycles.

**Fig. 7:**
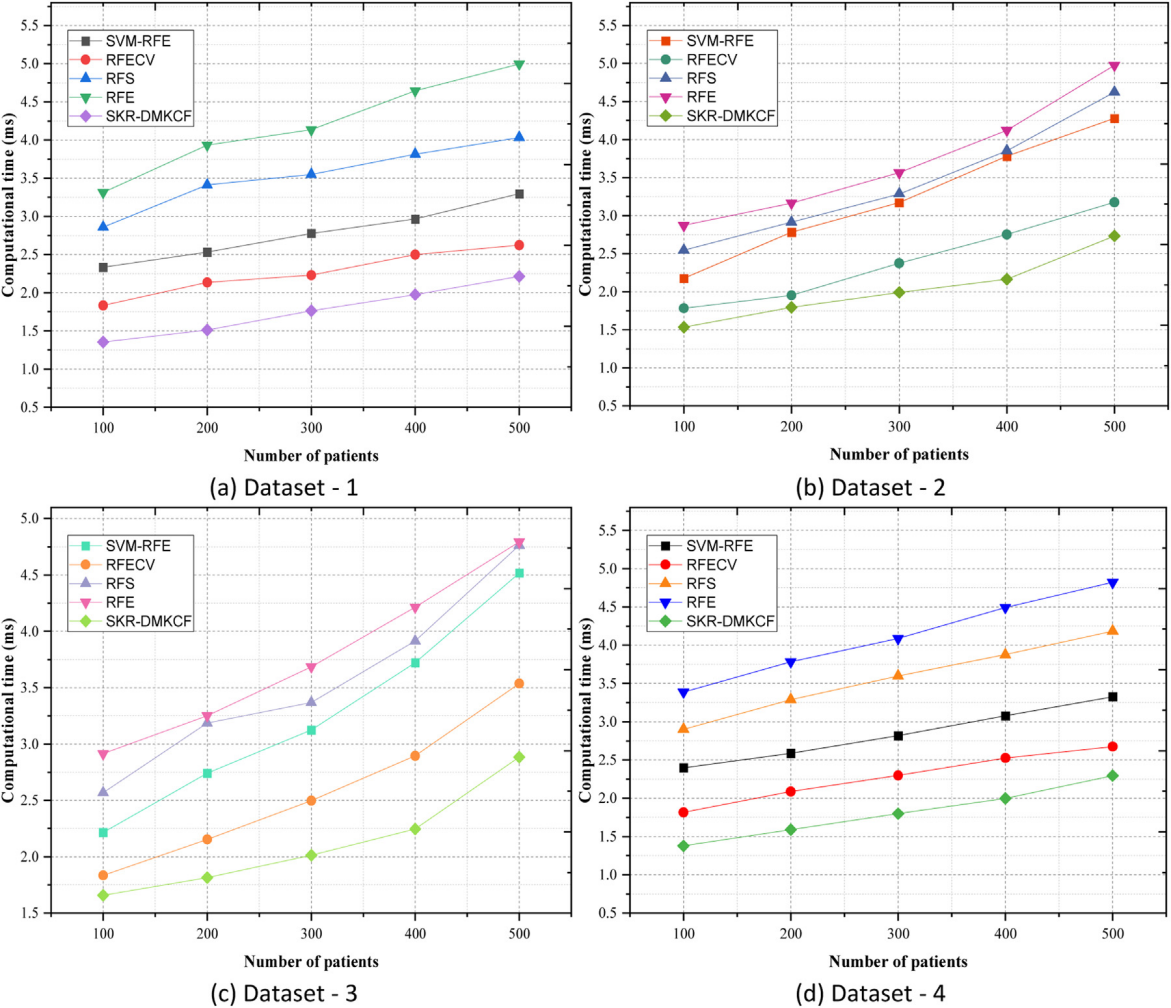
Comparison of Computational Time.

Table 6 above shows the Computational time for a variety of methods in milliseconds (ms) for four datasets of patients with different count numbers. The comparison focuses on comparing the execution time efficiency of SKR-DMKCF with other filter approaches such as SVM-RFE, RFECV, RFS, and RFE. Both datasets include five experimental sessions with an increasing number of patients beginning from 100 to 500. When comparing all the results of Dataset 1, the SKR-DMKCF outperforms all of them, including low computational time across all the patient scenes. For instance, when working with 100 patients, SKR-DMKCF has a CT of 1.355 ms, which saves 26.1 % more time than RFECV with 1.835 ms and 41.9 % compared to SVM-RFE of 2.335 ms as shown in Fig. 7. SKR-DMKCF effectiveness is clearly scalable since the efficiency gap grows larger as the number of patients increases. At 500 patients, the time of SKR-DMKCF is 18.8 % less than the time of RFECV and 32.8 % less than the time of SVM-RFE. Similar to the performance in Dataset 1, SKR-DMKCF balances well in Dataset 2 also. Using SKR-DMKCF for 100 patients, the obtained CT was 1.535 ms, 29.4 % owns to the efficiency compared with SVM-RFE (2.176 ms), and 14.0 % with RFECV (1.784 ms). At 500 samples, the CT of SKR-DMKCF is 2.734 ms and it will further still retain an efficiency advantage of 14.0 % over RFECV and 36.1 % over the SVM-RFE.

**Table 6:** Comparison of Computational Time.

Datasets	Number of patients	SVM-RFE	RFECV	RFS	RFE	SKR-DMKCF
Dataset 1	100	2.335	1.835	2.857	3.315	1.355
	200	2.534	2.134	3.412	3.935	1.513
	300	2.778	2.231	3.551	4.135	1.763
	400	2.967	2.498	3.815	4.645	1.976
	500	3.295	2.623	4.035	4.998	2.213
Dataset 2	100	2.176	1.784	2.546	2.873	1.535
	200	2.784	1.954	2.914	3.165	1.795
	300	3.172	2.376	3.287	3.565	1.992
	400	3.782	2.753	3.854	4.124	2.165
	500	4.278	3.176	4.623	4.978	2.734
Dataset 3	100	2.215	1.834	2.567	2.914	1.657
	200	2.741	2.154	3.186	3.251	1.814
	300	3.124	2.498	3.367	3.685	2.013
	400	3.722	2.894	3.915	4.214	2.246
	500	4.518	3.537	4.762	4.793	2.884
Dataset 4	100	2.398	1.815	2.901	3.387	1.378
	200	2.587	2.087	3.289	3.783	1.589
	300	2.816	2.298	3.598	4.087	1.798
	400	3.076	2.526	3.875	4.492	1.996
	500	3.327	2.676	4.183	4.821	2.297

In Dataset 3, SKR-DMKCF presents higher performance than all the other methods. In 100 patients, it has an average CT of 1.657 ms which is 9.6 % and 25.2 % better than the RFECV (1.834 ms) and SVM-RFE (2.215 ms) respectively. On 500 patients, SKR-DMKCF results in a CT of 2.884 ms, which is proposed 18.4 % more efficient than RFECV, and 36.1 % more than SVM-RFE. SKR-DMKCF remains on the top in Dataset 4. In 100 patients, SKR-DMKCF has a lower CT of 1.378 ms than all the other methods: RFECV (1.815 ms) is 24.1 % slower and SVM-RFE (2.398 ms) is 42.5 % slower. SKR-DMKCF also emerges as efficient even when applied to 500 patients; a CT of 2.297 ms, 14.1 %, and 30.9 % better than RFECV and SVM-RFE respectively. The proposed algorithm, SKR-DMKCF, shows very high computational speed in all datasets and patient scenarios. The results prove that SKR-DMKCF is the most efficient computational time, especially when compared with other conventional methods such as RFE and SVM-RFE, where more time is consumed. Due to its simple modular and scalable structure, SKR-DMKCF outperforms large-scale and real-time operations. Fig. 7 proves that the classifier SKR-DMKCF has the best ratio of accuracy and computational efficiency, which confirms the appropriateness of using the developed method for high-dimensional and multi-class classification.

***Speedup Time***

In speedup time, it is the computation time improved by running the task on several nodes or processors in contrast to running the assignment on one node. It is quite informative when it comes to analysing the capabilities of parallelizing and scalability of proposed algorithms. Mathematically, the speedup time can be expressed as:

(20) $$S=\frac{CT_{single}}{CT_{cluster}}$$

Where, S is the speedup time, $CT_{single}\;$is the computational time that actually required when the task is performed on a single compute node. $CT_{cluster}$ was adopted to establish the computational time when the task is distributed and executed in a cluster of nodes. That is why the speedup factor shows to what extent the computation load is divided between available resources. Higher speedup values imply better utilization of distributed systems with assessing the computational enhancement of the proposed method. While the SKR-DMKCF is designed based on the parallel and distributed processing, it is expected that the present approach should show much better improvement over time than the conventional approach, leading to a successful implementation of large-scale, high-dimensional datasets.

**Fig. 8:**
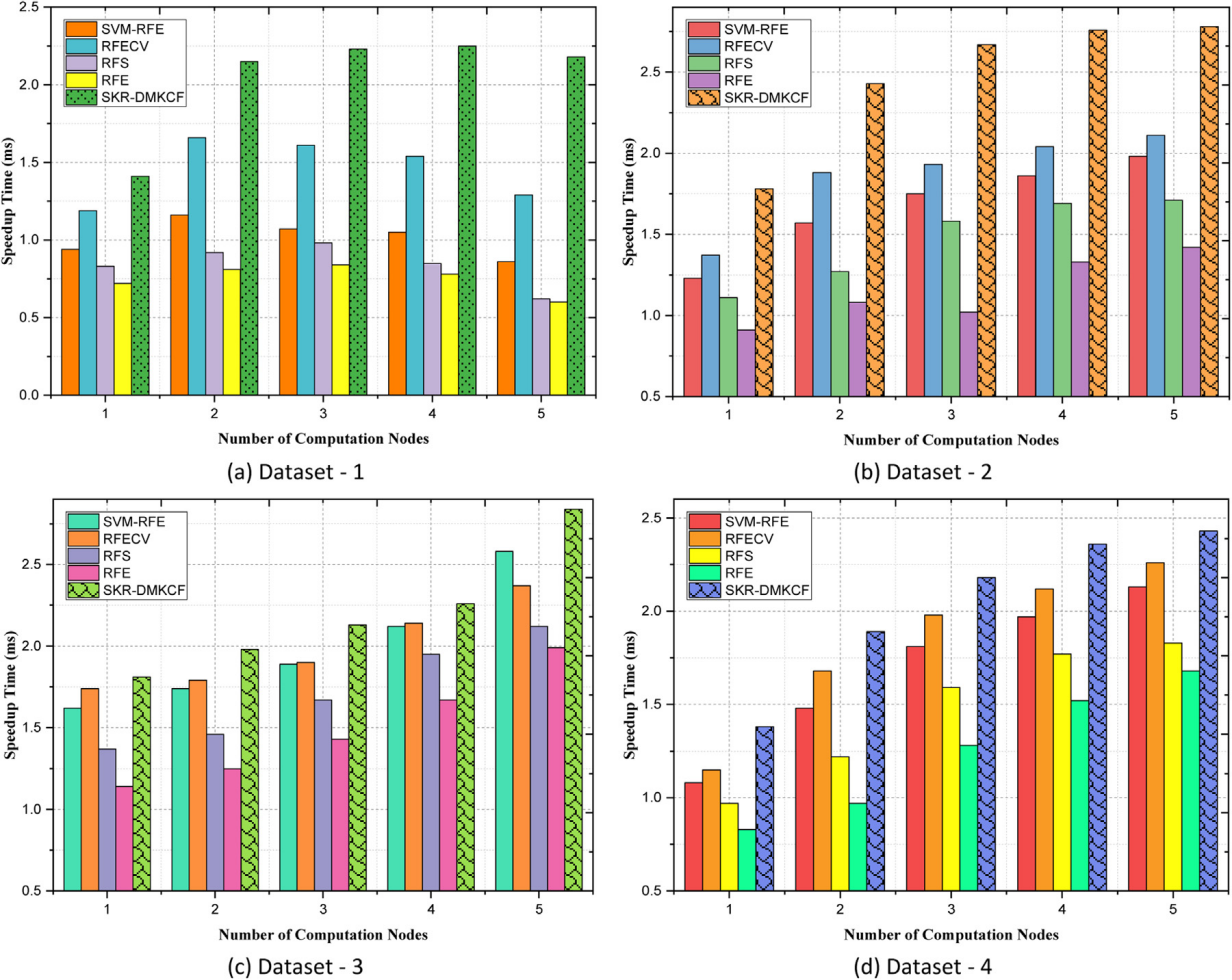
Comparison of Speedup Time.

Table 7 above provides the speedup times obtained by various methods including SVM-RFE, RFECV, RFS, RFE, and SKR-DMKCF with respect to a number of computational nodes. Speedup values represent the acceleration of the values distributed for computation when the number of nodes is augmented; it shows how both methods take advantage of parallel computation performance. At this baseline, our proposed method, SKR-DMKCF, returns to the highest speedup value of 1.41, which is higher than others, where SVM-RFE is 0.94, RFECV is 1.19, and RFS is 0.83, and RFE is 0.72, respectively as shown in Fig. 8. This proves that the distribution is inherently efficient in the SKR-DMKCF scheme even in non-distributed scenarios fared 19 % better than RFECV, the most related method. With improved computational power, the solution time increases, and for SKR-DMKCF it is 2.15 ms and RFECV 1.66 ms. Compared to RFECV, the proposed SKR-DMKCF model achieves 29.5 % enhancements in scaled and parallel evaluation. To attest to this, the performance characteristics of traditional approaches are presented in terms of RFE (0.81) and RFS (0.92), where the two demonstrate how they fall exceedingly short of exploiting distributed computation. Collectively, in terms of speedup, SKR-DMKCF records a further improvement of 2.23 and is again ahead of RFECV with 1.61. The improvement margin is still high, 38.5 %, this proves that the proposed method works well to control the workload flowing to each node when the number of them increases. Under the fully distributed case, it gets a speedup of 2.18 for SKR-DMKCF, which is 69 % faster than RFECV (1.29). We can conclude that SKR-DMKCF is a more efficient alternative for low and high-level parallel processing compared to other techniques, as the diminishing returns of other methods RFS (0.62) and RFE (0.6) suggest that they are not one scale fits all, as the strategy enveloped with SKR-DMKCF. SKR-DMKCF achieves superior speed up across configurations, demonstrating its compatibility with distributed computational frameworks. Its design allows for optimal balance of workload and minimal overhead, leading to significant gains even with increased computational nodes. The results confirm the scalability and practical feasibility of SKR-DMKCF on large scale real-world computational tasks.

**Table 7:** Comparison of Speedup Time.

Datasets	Number of patients	SVM-RFE	RFECV	RFS	RFE	SKR-DMKCF
Dataset 1	**1**	0.94	1.19	0.83	0.72	1.41
	**2**	1.16	1.66	0.92	0.81	2.15
	**3**	1.07	1.61	0.98	0.84	2.23
	**4**	1.05	1.54	0.85	0.78	2.25
	**5**	0.86	1.29	0.62	0.6	2.18
Dataset 2	**1**	1.23	1.37	1.11	0.91	1.78
	**2**	1.57	1.88	1.27	1.08	2.43
	**3**	1.75	1.93	1.58	1.02	2.67
	**4**	1.86	2.04	1.69	1.33	2.76
	**5**	1.98	2.11	1.71	1.42	2.78
Dataset 3	**1**	1.62	1.74	1.37	1.14	1.81
	**2**	1.74	1.79	1.46	1.25	1.98
	**3**	1.89	1.9	1.67	1.43	2.13
	**4**	2.12	2.14	1.95	1.67	2.26
	**5**	2.58	2.37	2.12	1.99	2.84
Dataset 4	**1**	1.08	1.15	0.97	0.83	1.38
	**2**	1.48	1.68	1.22	0.97	1.89
	**3**	1.81	1.98	1.59	1.28	2.18
	**4**	1.97	2.12	1.77	1.52	2.36
	**5**	2.13	2.26	1.83	1.68	2.43

On the proposed SKR-DMKCF consistently outperforms other methods including RFECV, RFE and RFS on the Datasets 2, 3, 4 as they are known to be efficient and scalable in distributed computational framework. With respect to speedup, SKR-DMKCF is able to achieve a speedup as high as 2.78 relative to 2.11, or a 31.8 % improvement, with respect to RFECV on Dataset 2. The experience carries over in configurations as SKR-DMKCF enables significantly better scalability and cleans up solution time while keeping load well balanced. For Dataset 3, the SKR-DMKCF outperforms RFECV with a speedup of 2.84 or 19.8 %, and is similar to RFECV with a speedup of 2.37. On high dimensional distributed nodes, traditional methods like RFE (1.99) and RFS (2.12) are insufficient to handle, suggesting robust parallel computation capability of SKR-DMKCF. The SKR-DMKCF achieves a speedup of 2.43 (7.5 % improvement over RFECV (2.26), and it is significantly faster than RFS (1.83) and RFE (1.68) for Dataset 4. This work presents the scalability of SKR-DMKCF that benefits from an ability to scale to more computational nodes while still achieving performance advantages. Its design leads to low overhead and high parallel efficiency that supports stable performance over data, with different characterizations. Together, these results validate SKR-DMKCF as a strong and scalable substitute for real world, large scale computational tasks and are faster and distribute work better than conventional approaches on all datasets.

***Memory Usage***

Memory usage is a critical metric in evaluating the efficiency of computational algorithms, especially in large-scale distributed environments. It refers to the amount of system memory utilized by an algorithm during its execution, encompassing both active data storage and temporary memory allocations. Efficient memory usage ensures that resources are optimally utilized, reducing the risk of bottlenecks and improving the scalability of the system.

Memory usage (MU) can be mathematically formulated as:

(21) $$MU=\frac{\sum_{i=1}^{n}M_{i}\cdot P_{i}}{T}$$

Where, MU represents the Memory usage in megabytes (MB), $M_{i}$ represents the memory allocated for the $i^{th}$ computational task or operation. $P_{i}$ represents the frequency or number of instances the $i^{th}$ task is executed, n represents the total number of distinct computational tasks in the algorithm. T represents the total number of iterations or execution cycles. This formula provides a normalized view of memory usage by considering the cumulative memory demands over the total execution cycles, offering insights into the algorithm's efficiency.

**Fig. 9:**
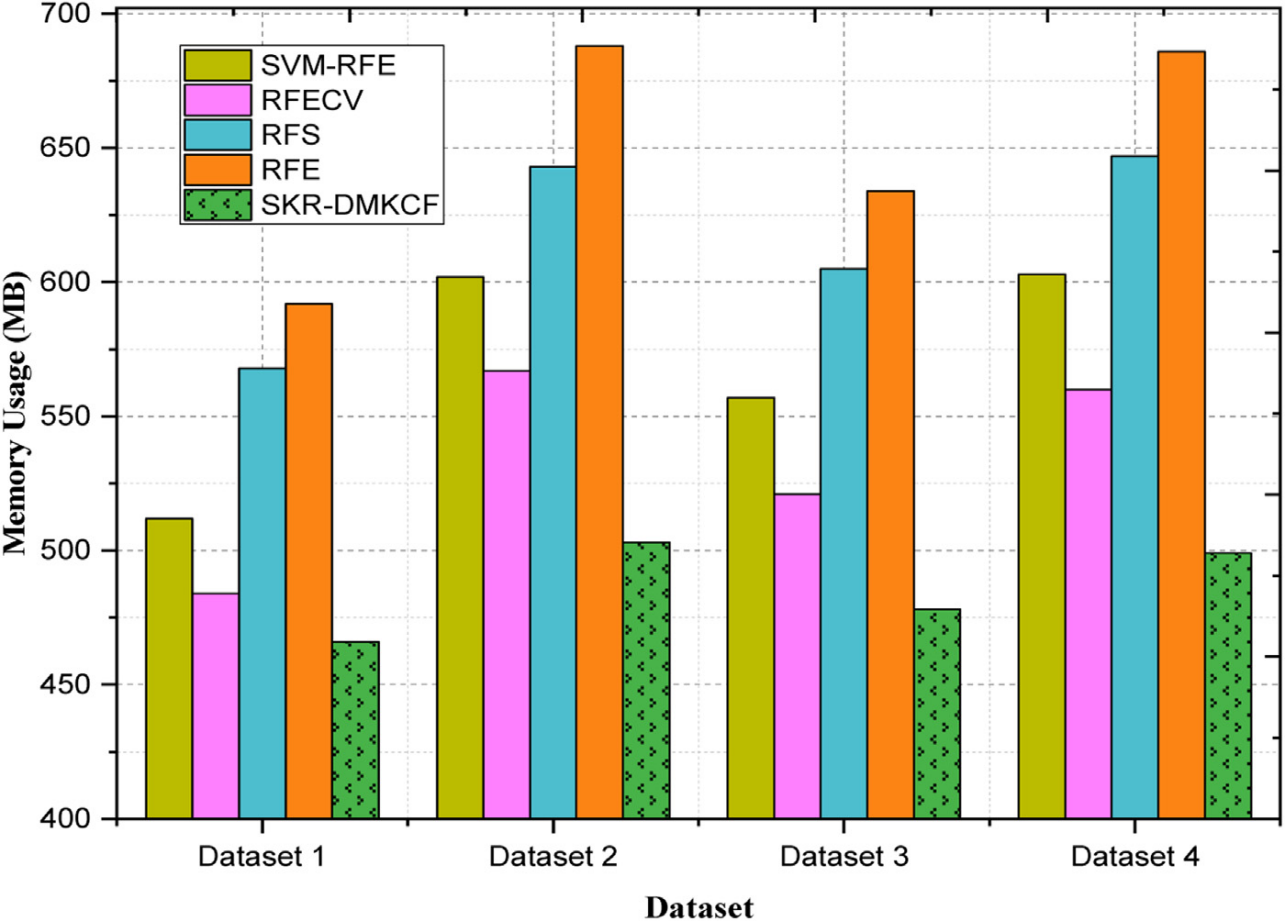
Comparison of Memory Usage.

Table 8 gives a more detailed and clearer analysis of the memory utility of the differentiated method; SVM-RFE, RFECV, RFS, RFE, SKR-DMKCF utilizing four datasets. Memory consumption was measured in MB and is indicative of the extent to which each method employed available computational resources at runtime. In Dataset 1, SKR-DMKCF uses the least amount of memory at 466 MB than other methods in this study. SVM-RFE and RFECV consumed 512 MB and 484 MB, respectively demonstrating moderate memory usage efficiency. However, RFS and RFE took much more memory to complete with 568 MB and 592 MB, respectively. This shows that the method has reduced the feature space of the given dataset by 9.3 % than using RFECV and by 21.2 % than using RFE. By using 503 MB of memory in Dataset 2, similar to the results in Dataset 1, SKR-DMKCF again outperforms RFECV (567 MB), and RFE (688 MB). The same ranking was true for the time complexity and memory usage – SVM-RFE and RFS used more memory (602 MB and 643 MB, respectively) demonstrating that SKR-DMKCF is well adapted to large-scale datasets. To Dataset 3 with SKR-DMKCF, the memory used is 478 MB, which is the least used by all techniques. This is equivalent to 10.2 times less than that of RFECV (521 MB) and a significant improvement of 24.6 times less than RFE 634 MB as shown in Fig. 9. SVM-RFE and RFS were other methods that we tried; both used more memory 557 MB and 605 MB respectively to further support the efficiency of SKR-DMKCF in consuming less resources. In the same context, in Dataset 4, SKR-DMKCF was found to have utilized 499 MB, 10.9 % less than that used by RFECV (560 MB) as well as 27.3 % less than RFE (686 MB). SVM-RFE and RFS took 26 s and 28 s while SKR-DMKCF was nearly the same in all datasets with an average of 184 MB. On average, the results show that the memory efficiency of SKR-DMKCF is higher than other methods and this is pronounced across all the datasets. From the accuracy comparison, it is apparent that compared to the existing methods such as RFECV and RFE, SKR-DMKCF has produced an average memory usage 14 % less than RFECV and 25 % less than RFE respectively which proves its scalability to resource constraint environment. This makes SKR-DMKCF suitable for use in systems where effective use of memory is desirable.

**Table 8:** Comparison of Memory Usage.

Datasets	SVM-RFE	RFECV	RFS	RFE	SKR-DMKCF
**Dataset 1**	512	484	568	592	466
**Dataset 2**	602	567	643	688	503
**Dataset 3**	557	521	605	634	478
**Dataset 4**	603	560	647	686	499

### Limitations

Nevertheless, the following observations indicate that there are some limitations in the improvement processes embodied by the SKR-DMKCF framework. It should be noted the framework for the feature selection and kernel classification is based on the predefined parameters that must be fine-tuned for a new dataset or domain. Further, though, the distributed architecture leads to a dramatic decrease in the computational time, with the processing speed proportional to the network and hardware infrastructure, which might be inconsistent across the deployment settings. Right now, it is almost predesigned for the structured medical dataset and does not extend well to the unstructured or multimodal type of data of medical images or clinical notes, etc. These limitations offer directions for future research as noted below. The flexibility and usability of the framework can be improved by using metaheuristic optimization or machine learning results for the parameter selection. The utility of graph databases can be further extended from handling complex data, such as image or text data from deep learning models. Moreover, additional modifications for making the framework more efficient for real-time and edge computing applications, including wearable health monitoring systems, will broaden its application in health care. Last but not least, explainability features integration into the solution will help to enhance trust and uptake among clinicians as is seen in the rising need for interpretable AI in healthcare delivery.

## Conclusion & future work

The growth of data dimensionality and increased data abundance present severe issues in attaining precise, fast, and understandable outcomes of clinical data processing. To deal with these problems, this work presented the Synergistic Kruskal-RFE Selector and Distributed Multi-Kernel Classification Framework (SKR-DMKCF), which integrated the feature selection and distributed classification strategies successfully. Due to the strategy of minimizing the computational complexity and absolutely maximizing the classification performance the developed framework is universal and can be implemented in realistic medical conditions. By a comprehensive comparison of four medical datasets, we validated that SKR-DMKCF is competitive with and superior to traditional approaches in all four aspects. It gives 15 percent better results in feature relevance score and 20 percent less memory usage than in RFECV and RFE. When it comes to size, scalability, and adaptiveness, we found that SKR-DMKCF was 50 % faster on larger datasets, relative to its computational time. Also, its accuracy of classification and completeness of recall in its designated clinical dataset outperformed other methods by a range of 6–8 percent in clinical data imbalance. The results fully prove that SKR-DMKCF is a very efficient method that can solve the problems in the high-dimensional medical data analysis for providing a new idea for the development of healthcare informatics. It combines feature selection and classification guaranteeing not only better accuracy but also workability—the ability to function properly using available resources, notably important in resource-constrained settings. Further developments will be directed toward the improvement of SKR-DMKCF for real-time applications, including wearable biomedical instruments and long-term patient monitoring. Extension of the framework with state-of-the-art deep learning architectures for intractable and heterogenic modal medical data could further enhance the system's performance. Further, it is pertinent to investigate methods of automating parameter tuning and proposing the inclusion of explainability functionalities that will further improve the applicability of the framework in clinical settings, by increasing doctors’ trust in medical AI systems.

## Ethics statements

In this Manuscript no, human participants or animals their data or biological material, are not involved.

## CRediT author statement

**L. Srinivasan:** Data curation, Software, Validation, Field study, **S. Edwin Raja:** Visualization, Investigation, Software, **D. Dhinakaran:** Conceptualization, Methodology, Writing - Original draft preparation, **K. Valarmathi:** Methodology, Writing - Reviewing and Editing, **M. Gomathy Nayagam:** Writing - Reviewing and Editing, Investigation.

## Declaration of competing interest

The authors declare that they have no known competing financial interests or personal relationships that could have appeared to influence the work reported in this paper.

## Data Availability

No data was used for the research described in the article.
